# Pediatric endocrine disorders: a review of intra-abdominal findings and appropriate imaging

**DOI:** 10.1007/s00247-026-06616-z

**Published:** 2026-05-02

**Authors:** Alankrit Shatadal, Kimberly K. Vidmar, David B. Allen, Teresa Chapman

**Affiliations:** 1 School of Medicine and Public Health, University of Wisconsin–Madison, Madison, United States; 2 Division of Endocrinology and Diabetes, Department of Pediatrics, University of Wisconsin–Madison, Madison, United States; 3 Department of Radiology, University of Wisconsin–Madison, 600 Highland Avenue, Madison, WI 53792 United States

**Keywords:** Adrenal function, Adrenal cortical tumors, Androgen insensitivity syndrome, Congenital adrenal hyperplasia, Müllerian anomaly, Pheochromocytoma

## Abstract

Endocrine disorders in pediatric patients often involve a variety of imaging modalities as part of a diagnostic workup or disease surveillance. This pictorial essay and review of the literature highlights adrenal and reproductive system imaging findings related to endocrine-related diseases. This review includes descriptions of normal adrenal gland anatomy and imaging characteristics, as well as the clinical presentations and diagnostic imaging of congenital adrenal hyperplasia and of benign and malignant adrenal masses. Additionally, normal reproductive tract development and puberty will be covered, followed by an overview of Müllerian anomalies, 46,XY gonadal dysgenesis, and polycystic ovarian syndrome. Differential diagnostic considerations, appropriate imaging protocols, and clinical management strategies will be discussed.

**Graphical Abstract:**

**Figure:**
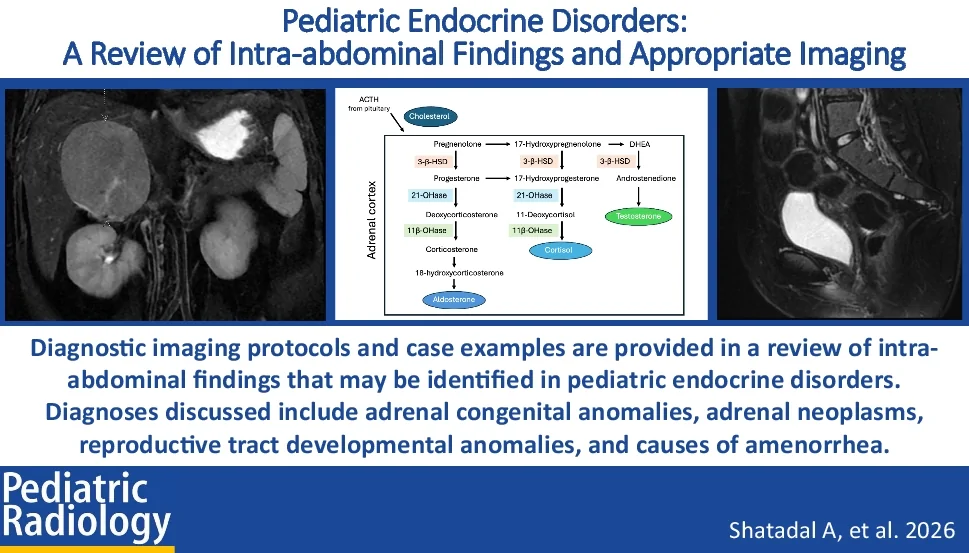

## Introduction

Pediatric endocrinologists specialize in diagnosing and treating hormonal disorders affecting growth, puberty, and metabolism in infants, children and adolescents. Evaluating abnormalities of the pituitary, thyroid, adrenal glands, and reproductive tract often relies on diagnostic imaging and consultation by pediatric radiologists. Abdominopelvic imaging, in particular, may be required for assessment of the adrenal glands and the reproductive tract. Adrenal abnormalities include congenital and neonatal disorders, such as congenital adrenal hyperplasia. Acquired adrenal diseases such as trauma and neoplasms may be identified at any pediatric age, from infancy through adolescence. Abnormalities of the reproductive tract in children may be evident at birth or may not be recognized until later in childhood or adolescence in the setting of either premature or delayed onset of puberty.

In this review, common and important abdominopelvic disorders addressed by both pediatric endocrinology and pediatric radiology will be described, with an emphasis on the endocrinology considerations and appropriate imaging modalities and protocols. Normal adrenal anatomy in the neonate is explained, followed by discussion of adrenal abnormalities including congenital adrenal hyperplasia and adrenal neoplasms. Reproductive tract embryology is reviewed. Abnormalities of the reproductive system discussed here focus on entities to explain amenorrhea, including Müllerian anomalies, differences of sex development, and polycystic ovarian syndrome. Demonstrative diagnostic imaging cases are provided.

### Adrenal gland anatomy and imaging

Adrenal glands are paired structures in the suprarenal spaces that are composed of two layers of different embryologic origins. The innermost adrenal medulla is formed by chromaffin cells from the neural crest and produces epinephrine (adrenaline) and norepinephrine (noradrenaline) [1], which affect energy availability, heart rate, and metabolic rate. The outer cortex derives from mesenchymal cells and synthesizes cortisol, aldosterone, and androgens, which control metabolism, sodium and water homeostasis, and responses to stress and inflammation [2]. These glands have an inverted Y-morphology or V-morphology and change in volume over time. In the fetus and neonate, the adrenal glands are 10 to 20 times the size of adult adrenal glands relative to body weight [3].

**Fig. 1:**
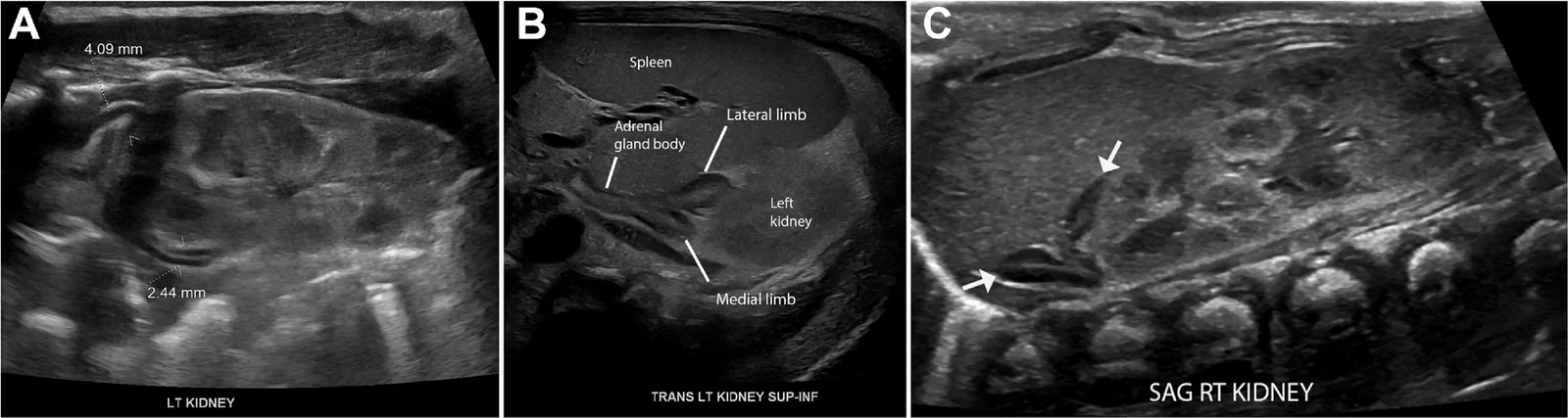
Normal adrenal glands in a 1-day-old newborn male infant imaged by ultrasound for renal function concerns, compared to normal adrenal glands in a 10-day-old infant’s renal ultrasound for antenatal diagnosis of urinary tract dilatation. **a** Longitudinal and (**b**) transverse views of the left adrenal gland and left kidney in a 1-day-old infant, showing normal architecture of the adrenal limbs, with hypoechoic cortex and thin echogenic stripe of the medulla. Calipers (**a**) denote thickness of each adrenal limb. **c** Longitudinal view of the right adrenal gland (*arrows*) and kidney in a 10-day-old male infant demonstrates a typical relative proportion of the adrenal gland length to renal length in infants

In infants and small children, adrenal glands are readily visible by ultrasound. Use of a high-frequency linear transducer provides optimal evaluation of the adrenal glands in neonates. On ultrasound, the normal neonatal adrenal gland has a characteristic thin echogenic core surrounded by a thick hypoechoic zone (Fig. 1). The gland rapidly decreases in length; in the newborn infant, the adrenal gland measures up to 23 mm in length, and this decreases to a maximum of 18 mm by 6 weeks of life [4, 5]. Newborn adrenal gland limb thickness measures less than 2.5 mm [5]. Subjectively, the neonatal adrenal gland is approximately one-third the length of the neonatal kidney (Fig. 1) [3, 5]. By age 1 year, the adrenal glands are similar in size and echogenicity to adrenal glands of an adult, with a subjective length that is less than 10% the length of the kidney [6].

**Fig. 2:**
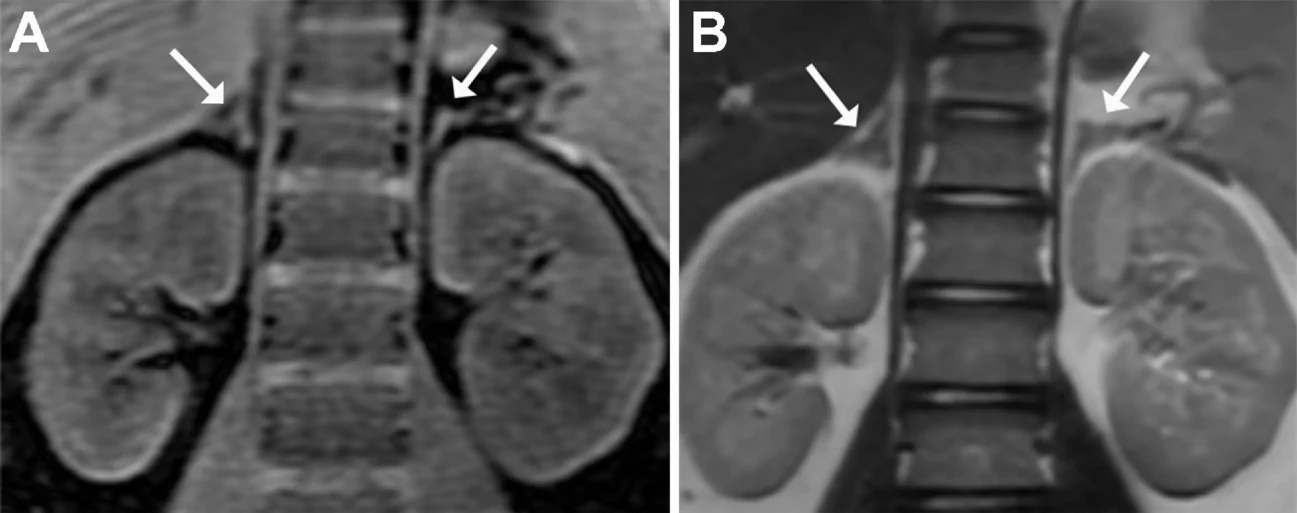
Normal adrenal glands on MRI of an 11-year-old female with secondary amenorrhea. Coronal (**a**) T1-weighted fat-suppressed and (**b**) T2-weighted single-shot fast-spin echo images show normal orientation and morphology of each adrenal gland (*arrows*), with an inverted Y-shape on the right and inverted V-shape on the left. Adrenal tissue is thin, without thickening, elongation, or mass. Signal is isointense to renal cortex on T1-weighted imaging and slightly hypointense to renal cortex on T2-weighted imaging

In the larger child, abdominal ultrasound may not provide sufficient anatomic detail of the adrenal glands due to their small size and deep position in the retroperitoneum. Adrenal imaging using abdominal magnetic resonance imaging (MRI) then becomes appropriate. Standard adrenal MRI protocols are summarized in Table 1 [7]. Normal adrenal glands in the older infant, school-age child, and adolescent demonstrate smooth contours, hypointense T2-weighted, isointense T1-weighted signal, and retroperitoneal fat between the adrenal glands and adjacent abdominal organs (Fig. 2). CT imaging can provide anatomic detail about the adrenal glands as well. Whereas adult CT adrenal protocols may include multi-phase imaging, this should be avoided in children because of greater concerns about the effects of ionizing radiation in the pediatric population and because there is no established advantage for multiphase CT imaging in pediatric adrenal masses, which will be discussed later. Pediatric single-phase contrast-enhanced CT is obtained in the portal venous phase [7]. Nuclear radiology exams including iodine-labeled metaiodobenzylguanidine (^123^I-MIBG and ^131^I-MIBG), 18F-fluorodeoxyglucose (^18^F-FDG)-positron emission tomography-computed tomography (PET/CT), and DOTA-DPhel-Tyr3-octreotate (DOTATATE) PET-CT may be relevant in evaluation of certain adrenal masses, discussed later in this article.

**Table 1:** Pediatric adrenal disorders that cause endocrine abnormalities and their key imaging features

Abdominopelvic pathology	Abdominopelvic imaging approach	Abdominopelvic imaging features	Clinical and endocrine features
Adrenal pathologies			
Congenital adrenal hyperplasia (CAH)	• Retroperitoneal ultrasound is the appropriate primary modality for assessment • A focused abdominal non-contrast MRI may be helpful in older and larger children/adolescents **MRI focused protocol (non-contrast):** Coronal abd/pel large FOV T2W SSFSE Coronal small FOV T2W FSE with FS focused on suprarenal space Axial T2WI FSE with FS Axial T1WI gradient echo	• Classic findings: enlarged adrenal gland with cerebriform morphology • May have stippled echogenicity on ultrasound • Some cases of CAH have no adrenal enlargement evident • Testicular adrenal rest tumors may arise in affected males: progress from multiple hypoechoic nodules with posterior acoustic shadowing to echogenic conglomerate mass near mediastinum testis	• Salt-wasting subtype presents with hyponatremia, hyperkalemia, hypoglycemia, hypotension shortly after birth • Simple virilizing subtype in the affected 46,XX fetus presents with clitoromegaly and fusion of labia majora • Non-classical subtype may present with premature adrenarche, hirsutism, cystic acne, irregular menses and infertility in females, accelerated linear growth, sporadic underproduction of cortisol when ill/stressed
Adrenocortical tumors	• Abdominal or retroperitoneal ultrasound as initial imaging modality to evaluate for a suspected mass in infants and young children • Abdominal MRI with and without contrast is appropriate where available (see below) • CT of the abdomen/pelvis may be the preferred cross-sectional modality at some centers: single portal-venous phase protocol is appropriate in children • Separate evaluation of the lungs with CT is recommended **MRI abdomen/pelvis full protocol:** Pre-contrast sequences: Coronal T2W SSFSE Axial T2W FSE w/and w/o FS Axial T1W IP/OP Axial T1W FSE w/and w/o FS Axial DWI echo planar Post-contrast sequences: Axial T1W arterial-phase FSE w/FS Axial T1W portal venous-phase FSE w/FS Axial T1W 5-min-delayed phase FSE w/FS Coronal T1W 3D FSE	• Adrenal adenomas: small, well-circumscribed, uniform attenuation or signal, no metastatic spread • Note: low signal on opposed-phase T1-weighted MRI sequences cannot exclude malignancy • Adrenal carcinoma: typically, large size (averaging 9.9 cm in maximum transverse diameter), heterogeneous enhancement, internal calcifications may be present. Metastatic spread evidenced by adenopathy	• Virilization including enlarged penis or clitoris • Accelerated skeletal maturation • In a minority of cases, Cushing syndrome (obesity, decreased linear growth) or hyperaldosteronism (hypertension, hypokalemia) • Association with genetic tumor predisposition syndromes
Pheochromocytoma and paragangliomas	• Abdominal or retroperitoneal ultrasound as initial imaging modality to evaluate for a suspected mass in infants and young children • Abdominal MRI with and without contrast is appropriate where available (as in adrenocortical tumors) • CT of the abdomen/pelvis may be the preferred cross-sectional modality at some centers: single portal-venous phase protocol is appropriate in children • Whole-body MRI screening for children and adolescents with germline mutations • Nuclear scintigraphy with ^123^I-MIBG or ^68^ Ga-DOTATATE PET-CT may be used for multifocal disease detection	• Increased vascularity on color Doppler • Round and well-defined with striking T2 hyperintensity and contrast enhancement with prolonged washout	• Hypertension, headache, palpitations, diaphoresis, abdominal pain, polydipsia • Associated with germline mutations
Ganglioneuroma	• Same approach as for adrenocortical tumors (above)	Definitive diagnosis cannot be made by imaging alone, though calcifications on US and permissive diffusion on DWI favor ganglioneuroma over ganglioneuroblastoma Features include: • Well-circumscribed ovoid mass • Attenuation on CT dependent on myxoid stroma content; whirling or patchy enhancement can be observed • Homogeneous T1-weighted hypointense signal and heterogeneous T2-weighted signal	• Often asymptomatic • Occasional catecholamine secretion causing flushing, tachycardia, hypertension
Ganglioneuroblastoma/neuroblastoma spectrum	• Same approach as for adrenocortical tumors (above) • CT chest is appropriate to evaluate for pulmonary metastatic disease • Nuclear scintigraphy with ^123^I-MIBG or ^18^F-FDG PET-CT for non-MIBG-avid disease is used to assess for metastatic disease	• Ill-defined mass, often originating from the adrenal medulla, displaying encasement or displacement of nearby vasculature • US with bright echoes and posterior acoustic shadowing if calcifications are present; anechoic cystic components • MIBG-avid or FDG-avid	• Abdominal distension, pain • Hypertension • Paraneoplastic syndromes are rare but include opsoclonus-myoclonus and VIP-induced diarrhea

*FDG*, fluorodeoxyglucose; *FS*, fat-saturation; *FSE*, fast-spin echo; *MIBG*, metaiodibenzylguanidine; *PET-CT*, positron emission tomography computed tomography; *SPGR*, spoiled dual gradient echo; *US*, ultrasound

### Congenital adrenal hyperplasia

**Fig. 3:**
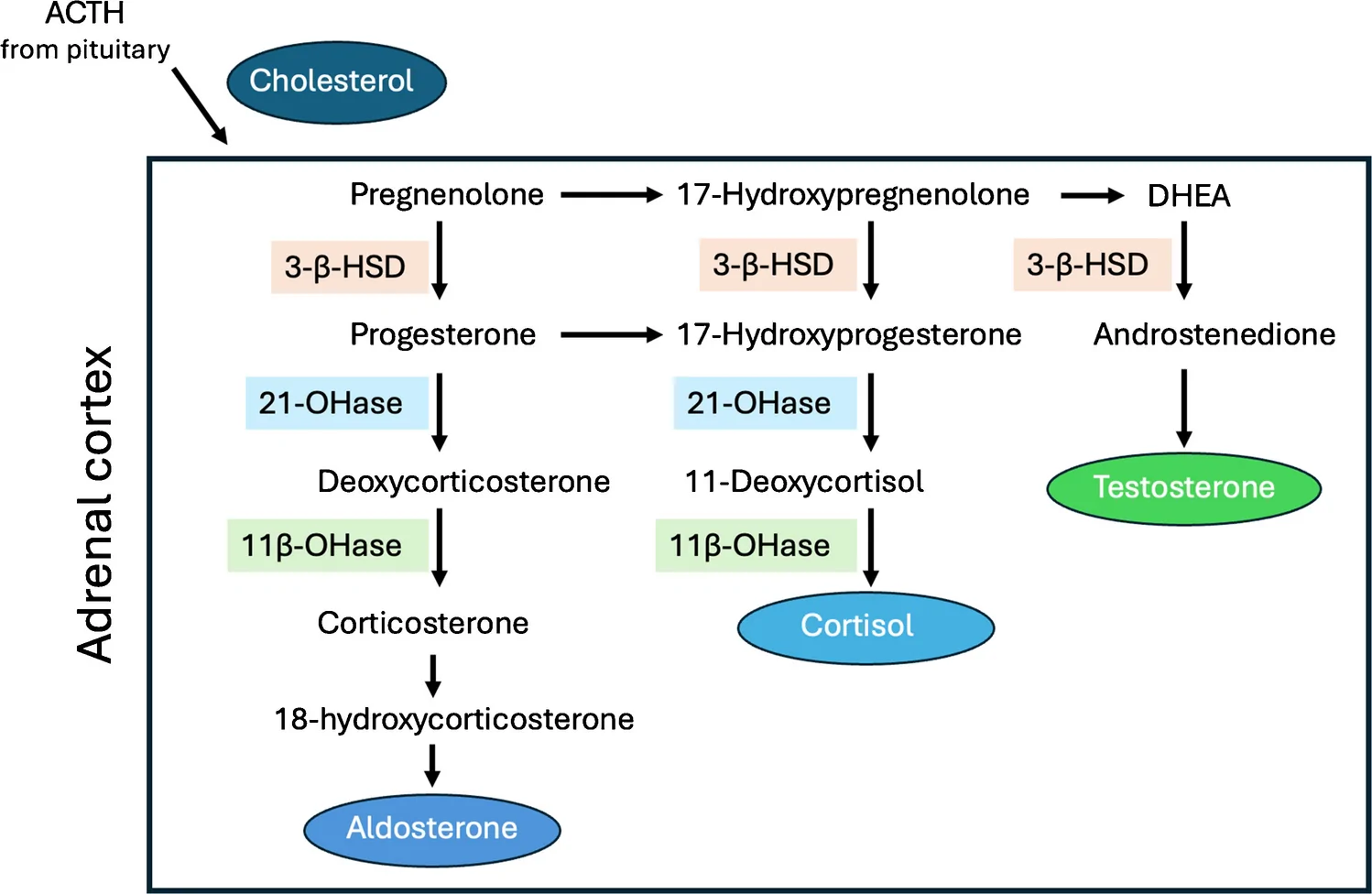
Steroidogenic biosynthesis pathway and relevant enzymes that may be deficient in congenital adrenal hyperplasia (*CAH*). The adrenocorticotropin hormone (*ACTH*) is synthesized by corticotroph cells of the anterior pituitary gland and binds to receptors on the surface of adrenal zona fasciculata cells producing cortisol. This stimulates the conversion of cholesterol into various steroid hormones in the adrenal gland. The steroidogenic pathway can be affected at different key steps depending on the type of enzyme deficiency: 21-hydroxylase (*21-OH*) deficiency is the most common cause of CAH, and 11beta-hydroxylase (*11β-OH*) and 3β-hydroxysteroid dehydrogenase (*3β-HSD*) are more rare enzyme deficiencies resulting in CAH. *DHEA*, dehydroepiandrosterone. Adapted from Fraga et al. [9]

Congenital adrenal hyperplasia (CAH) results from pathogenic variants in genes specific to the adrenal steroidogenic biosynthesis pathway [8, 9]. The most common form of CAH, accounting for approximately 95% of cases and seen in one in 15,000 live births, is caused by mutations of the *CYP21A2* gene which lead to 21-hydroxylase (21-OH) deficiency [8, 10]. The second most common form of CAH is 11β-hydroxylase (11β-OH) deficiency, which occurs in approximately one in 100,000 live births [10]. Other very rare enzyme defects, such as 3β-hydroxysteroid dehydrogenase type 2 (due to a *HSD3B2* gene defect), occur in fewer than one in one million live births [8]. Within the adrenal cortex, 21-hydroxylase is an essential enzyme for the synthesis of cortisol and aldosterone (Fig. 3). Without sufficient cortisol production, there is a loss of the normal negative feedback loop to the hypothalamus–pituitary gland axis, resulting in overproduction of adrenocorticotropic hormone (ACTH). This then results in overstimulation of the adrenal gland and resultant excess adrenal androgen production and adrenal hyperplasia [9].

**Fig. 4:**
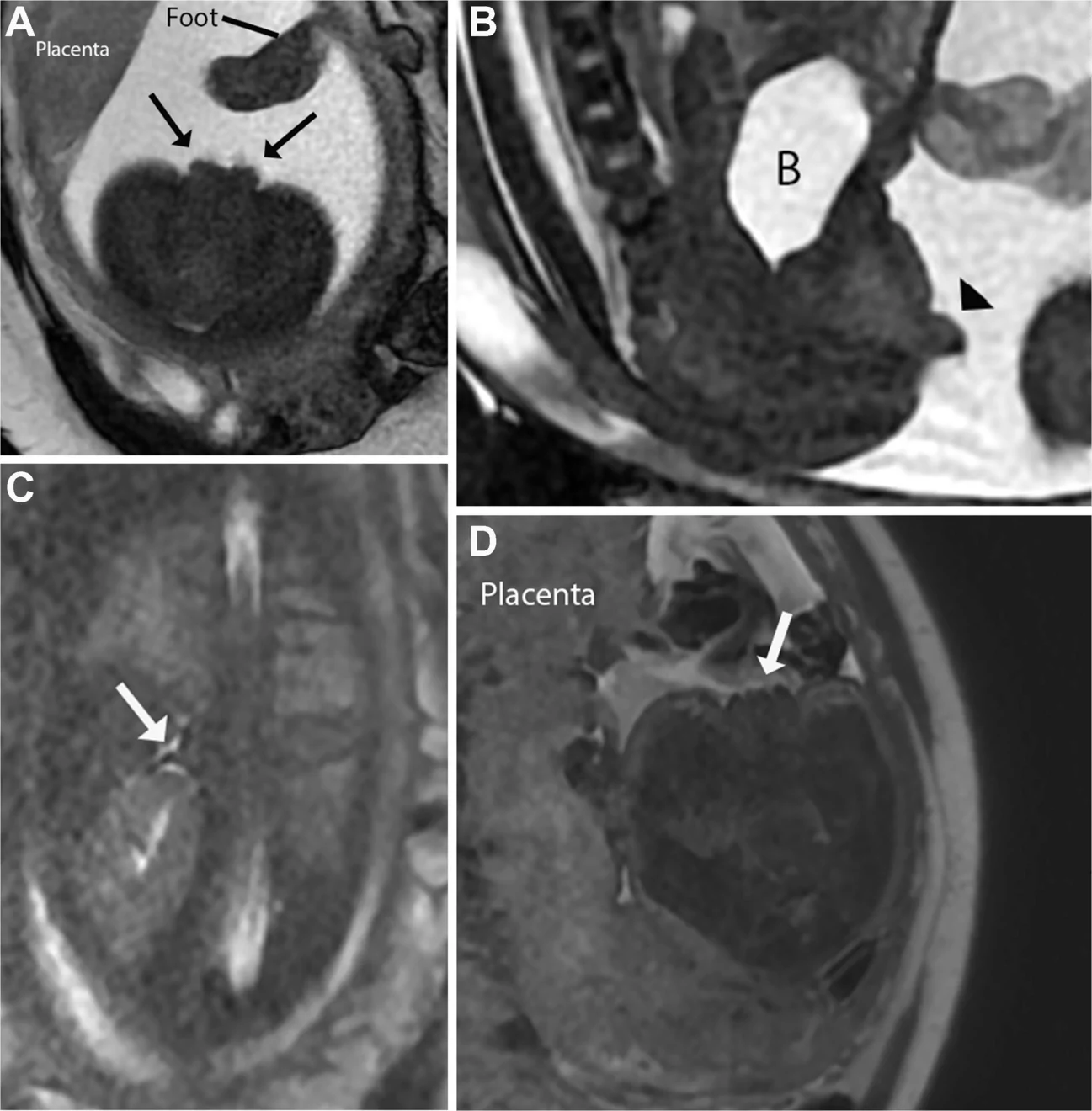
Atypical virilized genitalia identified by fetal MRI in a 46,XX fetus at 24 weeks gestation. Axial (**a**) and sagittal (**b**) balanced free precession T2-weighted images show labia majora (*black arrows*, **a**) and clitoromegaly (*arrowhead*, **b**). *B*, urinary bladder. **c** Coronal T2-weighted single-shot fast-spin echo of right adrenal gland appears normal (*white arrow*). No abnormality in the left suprarenal space (*not shown*) or elsewhere. Primary considerations include congenital adrenal hyperplasia or maternal sources for hyperandrogenism. **d** For comparison, normal appearance of fetal genitalia in a different 25-week GA 46,XX fetus. Axial T2-weighted SSFSE through the level of the perineum shows normal labia majora and minora (*white arrow*), without phallic structure

There are different phenotypes of CAH caused by 21-OH deficiency, with severity falling on a spectrum depending on the amount of enzyme activity [8, 9]. The classic form of CAH is the most severe due to critically deficient or absent enzyme activity. Classic CAH may be salt-wasting or simple virilizing, or a combination of both manifestations. Without urgent treatment after birth, cortisol and mineralocorticoid insufficiency can become life-threatening within the first few weeks of life. Adrenal crisis manifests with hyponatremia, hyperkalemia, hypoglycemia, and hypotension [8, 9]. Treatment involves replacement of the deficient cortisol and correction of the metabolic derangements. Meanwhile, virilizing effects seen in 46,XX fetuses affected by classic CAH are caused by accumulated steroid precursors upstream to the enzyme effects of 21-hydroxylase (Fig. 3), such as 17-hydroxyprogesterone (17-OHP) that are diverted into the adrenal androgen biosynthesis pathway, resulting in hyperandrogenism [8, 9]. Prior to birth, the excess production of adrenal androgens in affected 46,XX fetuses causes prenatal virilization of the genitalia at birth including clitoromegaly and fusion of the labia majora (Fig. 4) [8]. Although a prenatal suspicion of this disorder may arise because of virilized genitalia in 46,XX fetuses, there is typically no such prenatal clue in 46,XY fetuses affected by classic CAH; rarely, adrenal enlargement may be present and provides an indirect clue of the diagnosis [11]. For this reason, there is mandatory newborn screening for 21OH deficiency CAH in the USA.

The non-classic (milder) form of CAH is characterized by a later presentation of adrenal hyperandrogenism without cortisol deficiency under unstressed conditions. The presentation varies widely depending on the amounts of enzyme activity. In children younger than 10 years of age, the most common clinical presentation is premature adrenarche [12]. In adolescence or adulthood, females can present with irregular menses, acne, hirsutism, or infertility. Males with non-classic CAH may also present with premature adrenarche and/or linear growth acceleration, while others go undiagnosed because symptoms of adrenal androgen excess do not prompt medical attention [8, 12]. Although these individuals typically produce enough cortisol under normal conditions, they may not be able to produce enough cortisol during periods of increased stress, such as severe illness, injury, or surgery.

**Fig. 5:**
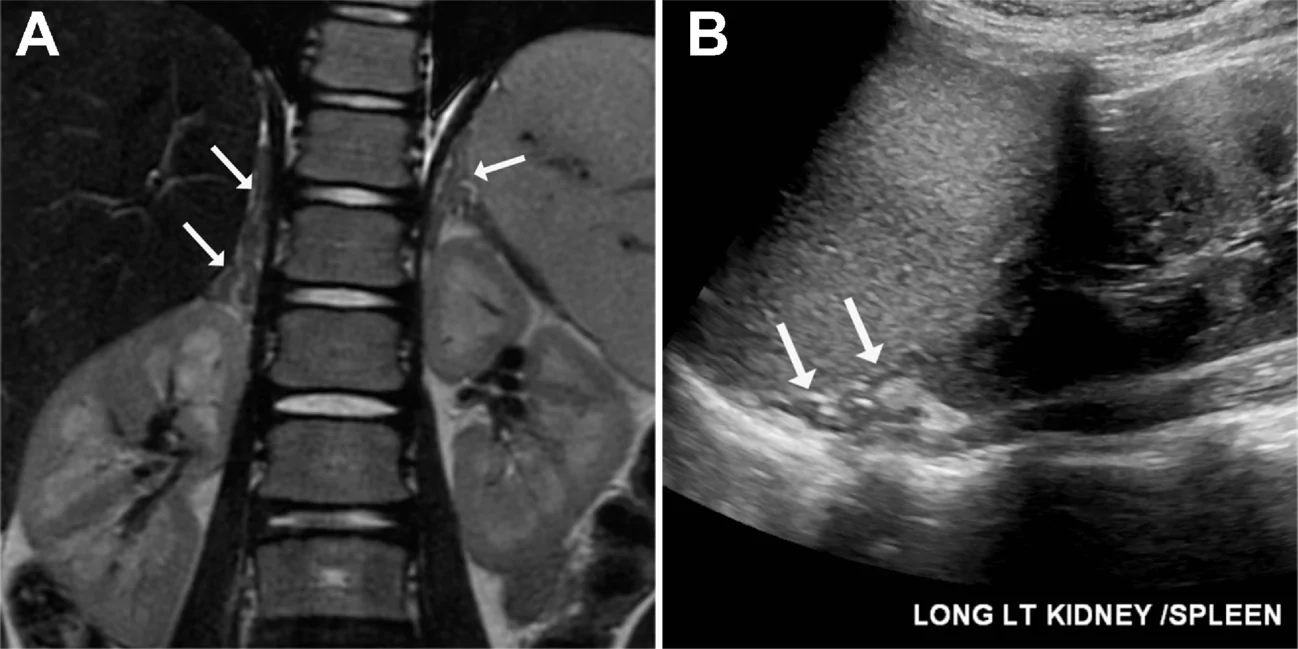
Congenital adrenal hyperplasia due to 21-hydroxylase deficiency in a 5-year-old female presenting with pubic hair at birth and later clitoromegaly. **a** Coronal T2-weighted abdominal MRI focused on the adrenal glands shows abnormally elongated and cerebriform morphology of bilateral adrenal glands (*arrows*). **b** Retroperitoneal ultrasound longitudinal view of left adrenal gland in same patient shows irregular morphology of the adrenal gland with stippled echogenicity (*arrows*)

**Fig. 6:**
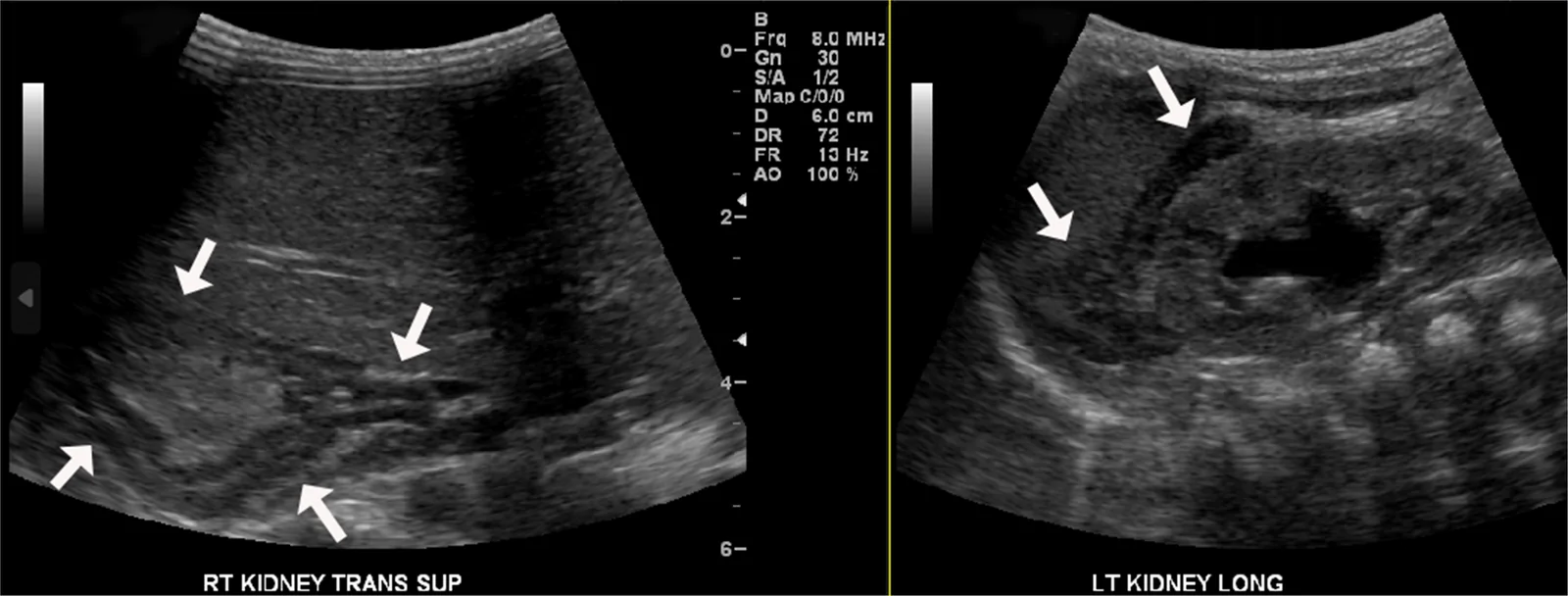
Congenital adrenal hyperplasia due to 3-beta hydroxysteroid dehydrogenase deficiency in a 4-day-old male infant imaged by ultrasound. Adrenal glands shown in transverse and longitudinal planes are larger than expected size of the adrenal glands relative to the kidney and also have a slight cerebriform morphology (*arrows*)

Abnormal enlargement of the adrenal glands in CAH may be evident on diagnostic imaging exams. The typical inverted Y shape is often preserved, but the gland will be enlarged either diffusely with maintained smooth margins or with a cerebriform morphology, characterized by tissue folding that resembles cerebral gyri (Figs. 5 and 6) [13]. Hyperplastic glands may also display a more stippled echogenicity (Fig. 5) [14]. Notably, only some CAH patients have discernible adrenal hyperplasia on diagnostic imaging.

Treatment for CAH requires daily glucocorticoid and, when needed, mineralocorticoid replacement. It is important that patients and families receive education on the need for stress dosing in times of illness, injury, or surgery, which warrant supraphysiologic glucocorticoid doses to adequately suppress ACTH production [15]. Particularly in cases where treatment has been inadequate, central precocious puberty may develop, as excess adrenal androgens can desensitize the hypothalamic-pituitary-gonadal axis, triggering premature activation of central puberty. This may require gonadotropin releasing hormone (GnRH) agonists to suppress endogenous puberty by reversibly downregulating the pituitary-gonadal axis. Management of patients with CAH requires multidisciplinary care, including input from endocrinology, genetics, urology, and health psychology. The latter can be especially important in addressing psychological stress issues regarding self-image, sexual orientation, and gender identity which occur with increased frequency in persons with CAH [16].

Dual X-ray absorptiometry (DEXA) scan and bone age radiography are additional imaging modalities used to screen for and/or monitor complications of CAH, which can include advanced bone age from excess androgens and decreased bone mineral density secondary to prolonged high-dose glucocorticoid exposure in the setting of over-treatment or frequent need for stress dose steroids [17]. There is no standard guideline for the timing or frequency of DEXA scanning in patients with CAH on steroids. Assessment of bone density is typically performed for patients subjected to prolonged periods of supraphysiologic glucocorticoid dosing or for patients on chronic steroids who have sustained a non-traumatic fracture. A diagnosis of pediatric osteoporosis requires both a bone mineral density *Z*-score less than −2.0 and a nontraumatic, clinically significant fracture history defined as a long bone fracture of lower extremities, vertebral compression fracture, or two or more long bone fractures of the upper extremities [18]. Pediatric endocrinologists rely on bone age evaluations in their effort to establish a balance between adrenal suppression and avoidance of excess glucocorticoid effect to help achieve satisfactory height growth. Chronic treatment with steroids and its effects on decreasing bone mineralization may affect bone age interpretation and, consequently, may make predicted heights inaccurate [19].

A potential complication of classic congenital adrenal hyperplasia in males is development of testicular adrenal rest tumors, seen in 20–40% of these patients [20]. Adrenal rests describe collections of steroidogenic cells responsive to adrenocorticotropin hormone (ACTH) that grow in the testes. Testicular adrenal rest tumors are benign bilateral masses with important implications for future fertility. Endocrine Society Clinical Practice Guidelines recommend screening with testicular ultrasound beginning in adolescence. There are no formal guidelines on frequency of screening, but many experts suggest every 1–2 years in asymptomatic males, or more often in symptomatic patients [10, 21].

**Fig. 7:**
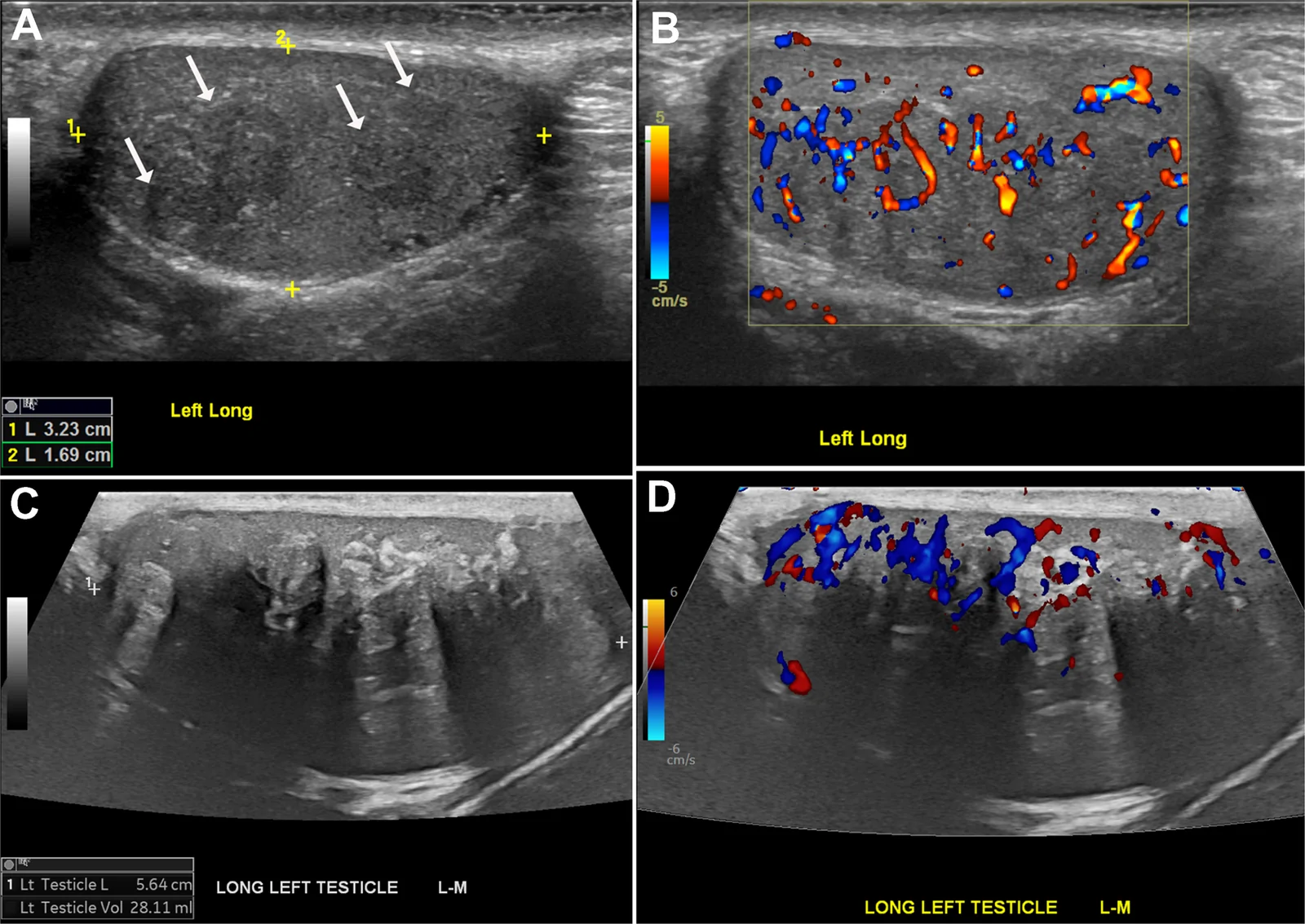
Testicular adrenal rest tumors in a 9-year-old male with history of congenital adrenal hyperplasia due to 3-beta hydroxysteroid dehydrogenase deficiency and testicular enlargement. Scrotal ultrasound shows (**a**) rounded scattered areas of hypoechoic tissue on grayscale image (*arrows*) and (**b**) hyperemia on color Doppler. **c**,** d** Repeat ultrasound performed later at age 15 shows (**c**) confluent areas of heterogeneity and echogenicity with posterior acoustic shadowing and (**d**) hyperemia on color Doppler, typical of testicular adrenal rest tumors

On ultrasound, these appear as ill-defined intratesticular hypoechoic and heterogenous masses, with marked hypervascularity. Serial ultrasounds over time show progression from a single hypoechoic nodule to multiple hypoechoic nodules to a single conglomerate mass near the mediastinum testis (Fig. 7) [22]. Testicular adrenal rest tumors gradually cause obstruction of the seminiferous tubules, which leads to peritubular fibrosis and ultimately infertility through obstructive azoospermia [20, 23]. There are no guidelines for prevention of testicular adrenal rest tumors; the first line of treatment is increased glucocorticoid treatment to adequately suppress ACTH. Surgery is not considered a treatment for infertility associated with these masses and should be reserved for cases presenting with significant pain and discomfort [22, 23]. Ultrasound is the preferred modality for detection and can reliably detect lesions too small to palpate. Although MRI should not be used as a primary modality, testicular adrenal rest tumors may be seen on MRI; their appearance is hypointense on T2-weighted sequences and hyperintense on T1 in comparison to testicular parenchyma, with well-defined margins [23].

### Adrenal masses

In children, both benign and neoplastic adrenal solid lesions occur. These abnormalities may be incidentally discovered during abdominal imaging performed for abdominal pain or other acute presentations, or they may be suspected based on a palpable abnormality in the infant or because of hypersecretion of adrenal hormones. The age of the child will be a primary factor to establish an appropriate differential diagnosis. In this section, benign and malignant pediatric adrenal masses that will be discussed include adrenocortical tumors, pheochromocytoma, and neuroblastoma and neurogenic tumors. Of important note, adrenal metastases occur in adults but are exceedingly rare in children.

#### Adrenocortical tumors

Adrenocortical tumors are masses derived from adrenocortical cells and include both adenomas and carcinomas. These two entities can be difficult to distinguish both on a histopathologic basis and by diagnostic imaging. Recent World Health Organization recommendations have attempted to clarify differentiating features between adenomas and carcinomas [24]. Histologically, straightforward adenomas are composed of lipid-rich clear cells in a variable mixture with eosinophilic/compact cells [24]. Clear-cut malignancy-related features that would be found in adrenal cortical carcinoma, such as vascular invasion, local invasion into adjacent structures, tumor necrosis, and increased mitotic activity, are not present in adrenal cortical adenomas [24]. Adrenal adenomas are uncommon in children and are characterized by heterogeneity of both histopathologic variants and functional activity [24].

Adrenal cortical tumors have a known bimodal pediatric age distribution, with an increased incidence among children younger than 3 years and older than 13 years [25]. When presenting in children, these tumors are almost always hormonally active, allowing for prompt laboratory testing and diagnostic imaging, in contrast to those in adults, which are hormonally active in less than half of adult cases [24, 25]. Pediatric adrenocortical tumors are more common in girls and most commonly result in virilization [25]. Affected individuals may present with pubic hair, accelerated growth, and skeletal maturation, an enlarged penis or clitoris, hirsutism, or acne due to excess androgen secretion. A smaller number of affected children (between 15% and 40%) present with Cushing syndrome, a condition of excess glucocorticoids, which manifests with hypertension, obesity, and decreased linear growth [25–27]. In a prepubertal child with Cushing syndrome, an adrenal cortical tumor is the most likely cause [28]. Less common clinical manifestations of these tumors in children include feminization or gynecomastia due to excess estrogens or signs of hyperaldosteronism such as hypertension and hypokalemia [26]. Adrenal cortical tumors may occur sporadically but are strongly associated with constitutional genetic abnormalities, particularly TP53-inactivating mutations, and IGF2 overexpression, and in tumor predisposition syndromes such as Li-Fraumeni syndrome and Beckwith-Wiedemann syndrome [25, 29].

**Fig. 8:**
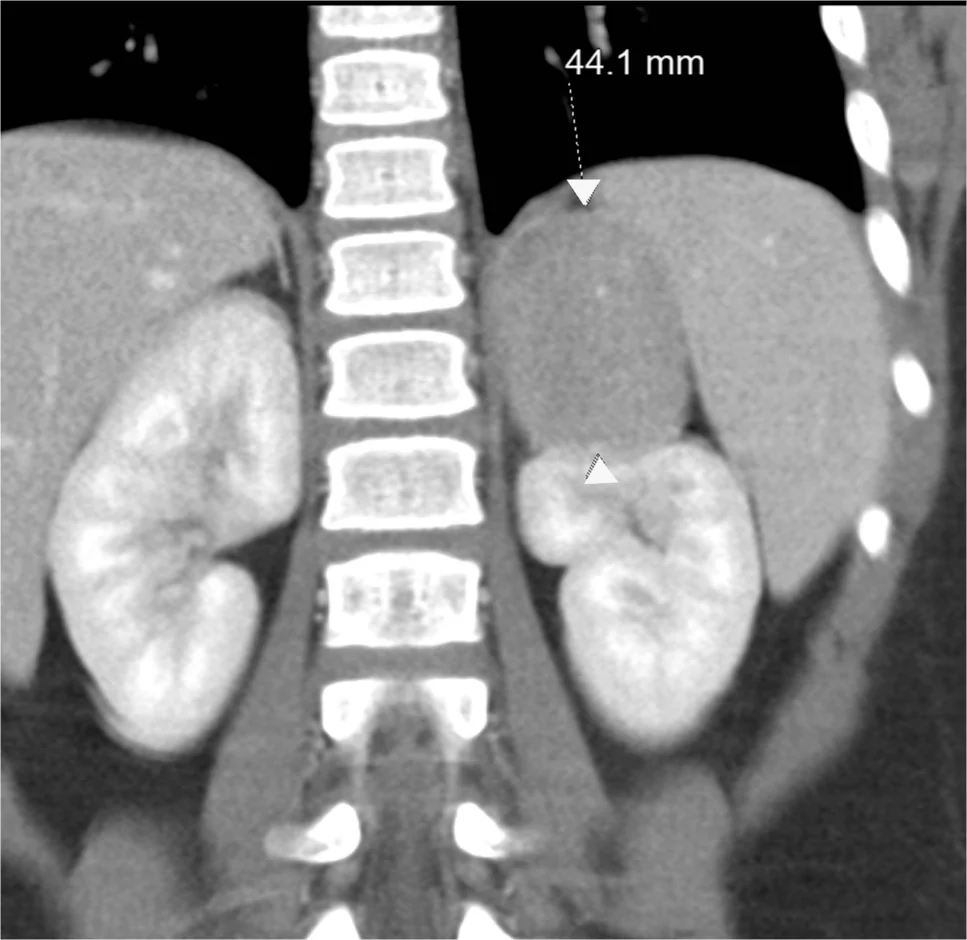
Virilizing adrenal cortical adenoma in a 4-year-old female who presented with pubic hair. Laboratory studies were notable for mildly elevated androstenolone sulfate at 89 mcg/dL (normal < 34) and elevated testosterone of 134 ng/dL (normal < 9). Coronal image reconstruction from a contrast-enhanced abdominal CT exam in portal venous phase shows a homogeneous, well-demarcated left suprarenal mass (*arrowheads*) maximally measuring 4.4 cm in craniocaudal height, consistent with functional adenoma in this clinical setting

**Fig. 9:**
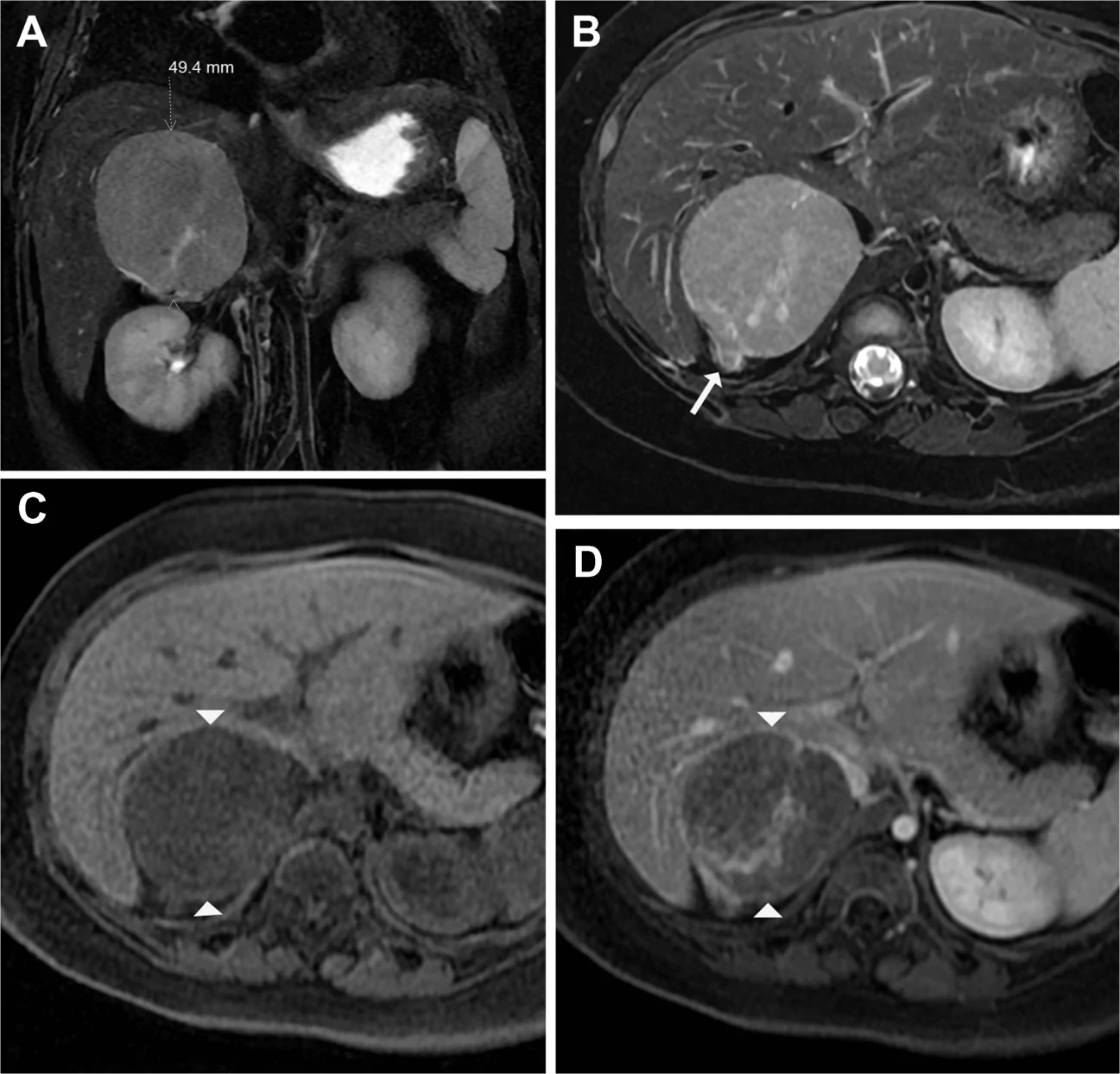
Adrenal cortical carcinoma in a 9-month-old female who presented with Cushing syndrome and hyperandrogenemia. **a** Coronal and (**b**) axial T2-weighted fat-suppressed images through the abdomen show a well-demarcated mass maximally measuring 4.9 cm in diameter with some internal heterogeneity. Of note, at the posterior margin of the mass, there is a small lobule of tissue extension with trace adjacent fat-stranding consistent with extracapsular extension (*arrow*), a finding concerning for aggressive tumor behavior and carcinoma. **c **Axial pre-contrast and (**d**) post-contrast T1-weighted fat-saturated images show rim enhancement and minimal internal linear and nodular enhancement (*arrowheads* indicate mass margins)

There are no published imaging criteria for differentiating adrenal adenomas from carcinomas in children. Certain diagnostic imaging tools used in adults, such as contrast washout thresholds on CT imaging and dropout of signal on out-of-phase T1-weighted MRI, cannot reliably exclude malignancy in pediatric cases [7]. On imaging, adrenal adenomas tend to be well-circumscribed and uniform in attenuation, without findings concerning for nodal metastasis (Fig. 8). MRI may show low signal on opposed-phase T1-weighted imaging compared to in-phase, but radiologists cannot rely on this to predict a tissue diagnosis [7]. Extracapsular spread of disease and nodal metastases are important imaging features that contribute to staging (Fig. 9). Evaluation of lungs for pulmonary metastatic disease using CT is appropriate upon identification of an adrenal cortical tumor. In a retrospective study comparing the diagnostic imaging findings of nine adrenocortical adenomas and 15 carcinomas in children [30], features shown to occur more frequently with adrenal cortical carcinomas included large size (mean maximum transverse diameter of 9.9 cm in carcinomas, compared with a mean of 4.4 cm in adenomas), heterogeneity on post-contrast imaging, intratumoral calcification, and metastatic disease at the time of diagnosis.

The mainstay of treatment for pediatric adrenal cortical carcinoma is complete surgical resection and lymph node dissection when nodal spread is detected. Chemotherapy is added for stage 3 and 4 disease [26, 27]. The main factor associated with a lower overall survival rate is metastasis at diagnosis. In patients with non-metastatic ACC, increasing age has been associated with lower overall survival [25, 27]. Surgical treatment is curative for benign adenomas [25, 27].

#### Pheochromocytoma and paraganglioma

A pheochromocytoma is a tumor that originates from adrenomedullary chromaffin cells and produces varying levels of catecholamines including epinephrine, norepinephrine, and dopamine. A paraganglioma is also a catecholamine-secreting tumor that arises from extra-adrenal chromaffin cells in the sympathetic paravertebral ganglia of the neck, chest, abdomen, or pelvis. Following current Endocrine Society guidelines, these two tumors are collectively referred to as pheochromocytomas and paragangliomas (PPGLs) [31]. Because of the catecholamine secretion, presenting symptoms may include sustained hypertension, headache, palpitations, profuse sweating, abdominal pain, nausea, vomiting, polyuria, and increased thirst [32, 33]. PPGLs are rare in children and adolescents and are likely to be associated with germline mutations, with up to 80% of these tumors diagnosed in children with a genetic mutation syndrome, such as multiple endocrine neoplasia type 2 (MEN 2), neurofibromatosis type 1 (NF1), von Hippel–Lindau disease, and the familial paraganglioma syndromes (*RET* mutation, succinate dehydrogenase enzyme mutations, *TMEM127* mutation, *MYC*-associated factor X mutation, and NF1) [32, 34]. Asymptomatic tumors may be identified because of recommended annual screening guidelines in high-risk populations, which includes whole-body MRI [35–37]. Compared to adults, malignancy of PPGLs is more common in pediatric patients, particularly with paragangliomas and tumors larger than 6 cm in size at presentation [35, 36]. Published pediatric series of PPGLs document rates of malignancy in children ranging from 14% to 47% [38–41]. It is known that succinate dehydrogenase (*SDHB*) germline mutations confer the highest risk for metastatic disease in children [42]. Separating out pheochromocytomas from all PPGLs, most pediatric pheochromocytomas (86–97%) are benign. Malignant behavior is more reliably determined on imaging based on the identification of local invasion and/or metastasis rather than using histologic analysis. Common sites of metastases are the lymph nodes, bones, liver, and lungs [34, 39].

**Fig. 10:**
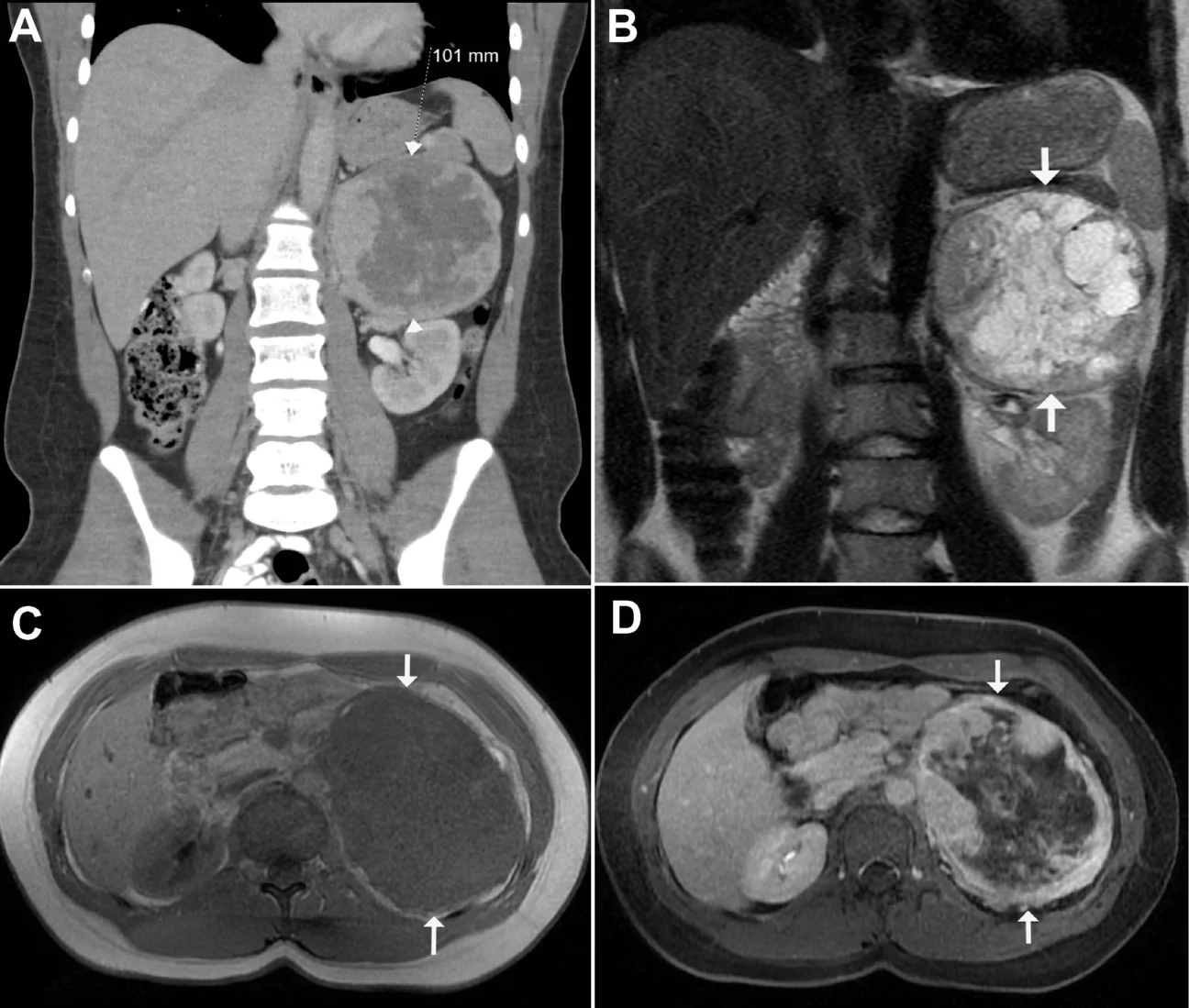
Pheochromocytoma in a 17-year-old female with shortness of breath and palpitation but normotensive. **a** Contrast-enhanced abdominal CT shows a large 10-cm left suprarenal mass with peripheral nodular enhancement and central low attenuating solid tissue (*arrowheads*). **b ** Coronal T2-weighted and (**c**) axial T1-weighted fat-suppressed pre-contrast and (**d**) post-contrast images show evidence of internal cystic components that are a frequent imaging feature of pheochromocytoma (*arrows* indicate mass margins)

As with all pediatric adrenal imaging, ultrasound is often the initial diagnostic imaging modality, but a normal exam does not exclude the presence of PPGL. When visualized, a pheochromocytoma is typically a round or oval solid mass in the suprarenal space with variable echogenicity and increased vascularity on color Doppler [7, 14]. MRI is the imaging of choice for pediatric patients and is used for screening of cancer-predisposition syndromes because it does not involve ionizing radiation [37]. On MRI, pheochromocytomas are round, well-defined masses with striking T2-weighted hyperintensity and avid contrast enhancement with a prolonged washout phase [7, 14] (Fig. 10). Computed tomography (CT) is recommended as a lesser imaging modality choice due to the associated risks of ionizing irradiation, although CT provides the advantage of outstanding spatial resolution and the ability to evaluate for pulmonary metastatic disease. On contrast-enhanced CT, a pheochromocytoma may be homogeneous or heterogeneous, but is typically the latter, attributable to intratumoral cystic components and occasional calcifications (Fig. 10).

Nuclear scintigraphy has a role in PPGL cases when there is suspicion for multifocal or metastatic disease. ^123^I-MIBG scintigraphy is useful, as norepinephrine receptors in PPGL tumors result in MIBG uptake allowing for identification on whole-body single photon emission computed tomography combined with CT (SPECT-CT). ^123^I-MIBG scintigraphy has a high specificity for detecting catecholamine-secreting tumors (84%); however, its sensitivity is not as high as PET-CT using ^68^ Ga-DOTATATE, which has been approved for localization of somatostatin receptor-positive neuroendocrine tumors in adult and pediatric patients. This technique provides a useful tool for detection of metastatic disease with improved diagnostic accuracy and superior sensitivity of approximately 96% [7, 43].

The primary therapy for both benign and malignant pediatric PPGLs is surgical resection, which is curative in benign cases [35]. Of note, complete surgical resection has been shown to be more successfully achieved with pheochromocytomas than with paragangliomas [36]. For unresectable or metastatic disease, options include radiation therapy and chemotherapy, although the evidence for pediatric use of these treatment modalities is limited [44].

#### Ganglioneuroma/ganglioneuroblastoma/neuroblastoma

The International Neuroblastoma Pathology Classification (INPC) describes four peripheral neuroblastic tumor subtypes falling on a spectrum of most aggressive features to benign behavior: neuroblastoma, ganglioneuroblastoma-nodular subtype (GNB-N), ganglioneuroblastoma-intermixed (GNB-I), and ganglioneuroma [45]. A ganglioneuroblastoma is characterized histopathologically as a stroma-rich tumor with a mixture of neuroblasts and ganglion cells in varying proportions. The GNB-N subtype has more immature components, with macroscopically visible nodules of neuroblastoma [46]. GNB-N tumors can be approached like a neuroblastoma for evaluation and prognostication, as will be discussed here. In contrast, because of the mature cellular behavior of GNB-I, diagnosis and management essentially match those of a ganglioneuroma [47, 48].

**Fig. 11:**
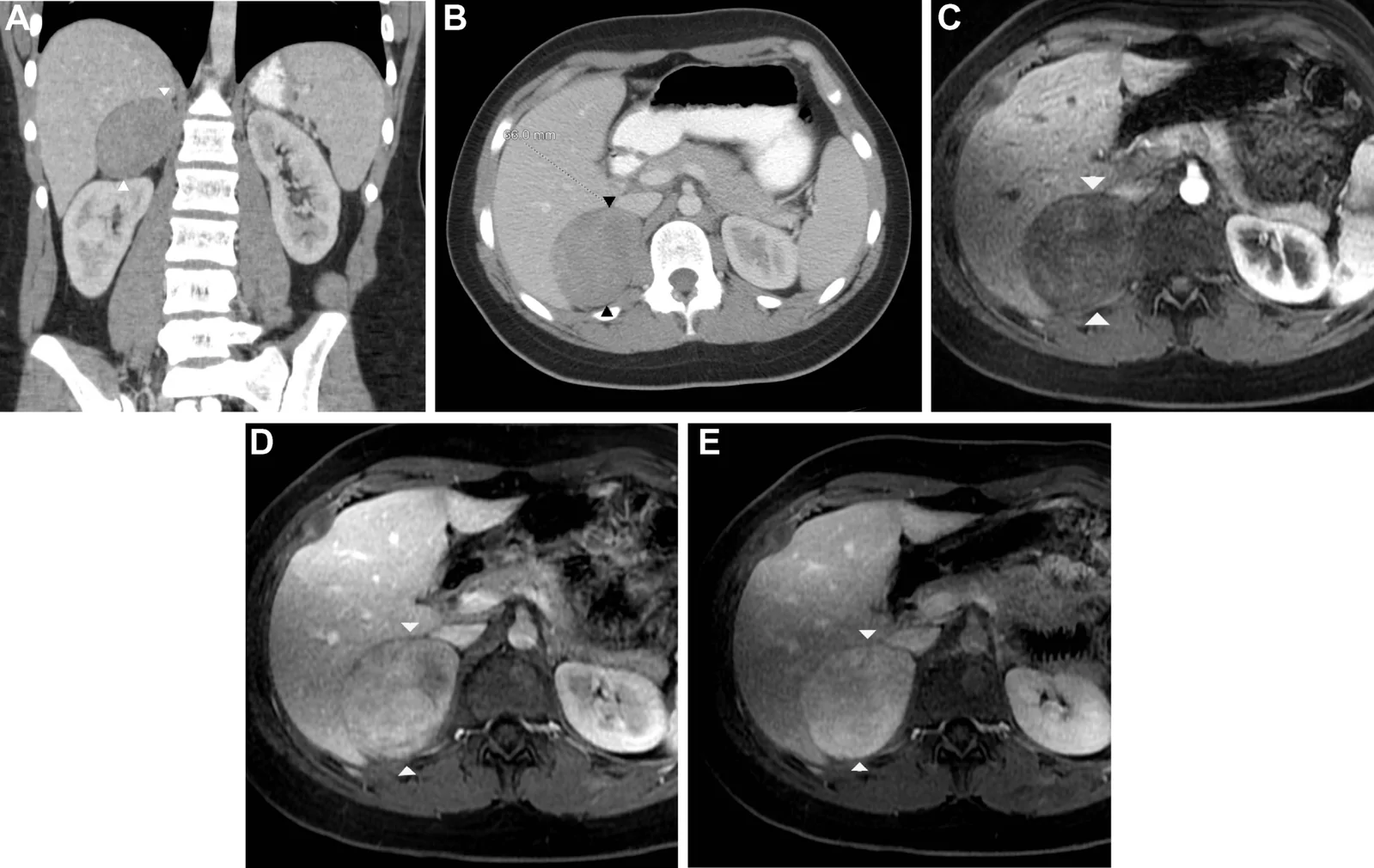
Ganglioneuroma identified incidentally in a 13-year-old female presenting with abdominal pain. **a**, **b** Contrast-enhanced CT images of the abdomen reconstructed in (**a**) coronal and (**b**) axial planes show a right suprarenal well-defined mass isodense to muscle (*arrowheads*), maximally measuring 6 cm. **c**-**e** Post-contrast T1-weighted fat-suppressed MRI in (**c**) arterial phase, (**d**) portal venous phase, and (**e**) 5-min delayed phase demonstrate progressive enhancement of the lesion over time (*arrowheads *indicate mass margins)

Ganglioneuromas are benign neurogenic tumors, consisting of mature ganglion cells within a spindle cell and myxoid stroma. Individuals with this tumor may present with pain or respiratory distress, although frequently these tumors are asymptomatic with incidental discovery on diagnostic imaging done for unrelated reasons [48]. Rarely, tumor secretion of catecholamines can cause flushing, tachycardia, and hypertension [14]. Patient age at diagnosis ranges from preschool age through adolescence (mean age of 7.5 years to 8.4 years) [48, 49]. Tumor size at presentation is typically large, at least 5 cm in maximum dimension [7, 49]. On ultrasound, ganglioneuromas in the suprarenal space are well-circumscribed with solid homogeneous hypoechoic tissue. Calcifications are reported in 35–50% of these masses and will appear as scattered punctate or lobulated shadowing echogenicities [49, 50]. On CT and MRI, these tumors are round or oval-shaped with well-defined margins (Fig. 11) [49]. Attenuation of the tumor on CT depends on the stromal composition and will be lower if there is a large amount of myxoid stroma present [49]. On MRI, ganglioneuromas demonstrate homogeneous T1-weighted hypointense signal and heterogeneous T2-weighted signal intensity (Fig. 11). The post-contrast enhancement on both CT and MR images progresses over time on multiphase images and may show a whirled or patchy pattern [7, 49]. These diagnostic features are attributable to the mixture of myxoid stroma and interlacing bundles of longitudinal and transverse Schwann cells and collagen fibers [7]. MIBG avidity may be seen in ganglioneuromas, and serum and urine catecholamine metabolites may be present in these patients [51]. In the absence of metastatic disease, imaging alone cannot differentiate a ganglioneuroma from a ganglioneuroblastoma or neuroblastoma, although permissive diffusion on diffusion-weighted imaging is supportive of a ganglioneuroma with low cellularity [13, 52]. Treatment is usually surgical resection alone with an excellent prognosis [47, 53].

Neuroblastoma is the most common extracranial solid malignancy in children and originates from the developing peripheral sympathetic nervous system, specifically from primitive neural crest cells undergoing defective neuronal differentiation [54–56]. The most common site of neuroblastoma is the adrenal medulla, accounting for approximately 50% of neuroblastoma cases, followed by the posterior mediastinum and then extra-adrenal intra-abdominal sites along the sympathetic chain [57]. Neuroblastoma is predominantly a cancer of small children, tending to present before age 5 years and with a median age at diagnosis of 19 months [56]. It is the most commonly diagnosed cancer in infancy, with approximately 40% of cases diagnosed within the first 3 months of life [57, 58]. Prognosis is highly varied, ranging from spontaneous regression to metastatic progression despite aggressive therapy. Of note, prognosis is significantly better in infants diagnosed with neuroblastoma compared to children over 1 year of age [59, 60]. Over that age, the 5-year survival rate diminishes [56–58]. The most common sites of metastasis include the lymph nodes, bone, bone marrow, liver, and skin [56, 57, 61].

Clinical symptoms are diverse and vary depending on the anatomic location of the tumor. Abdominal masses may be palpable on clinical exam in the asymptomatic child or may manifest with constipation, abdominal distention, abdominal pain, renin-induced hypertension due to renal vessel compression by tumor, or symptoms secondary to spinal canal involvement [57, 61]. A subset of children with neuroblastoma and with ganglioneuroblastoma present with a paraneoplastic syndrome, the most common being opsoclonus myoclonus syndrome (OMS), occurring in 2–4% of cases at presentation [62]. OMS is characterized by involuntary eye movements, muscle jerks, and ataxia. This entity presumably occurs because of autoantibody production against tumor antigens that cross-react with normal neural tissue [63, 64]. This syndrome is observed much more frequently in low-risk neuroblastoma compared to high-risk neuroblastoma, and more specifically with tumor types that exhibit a Schwannian stroma-rich background [65]. A less common paraneoplastic syndrome seen with neuroblastoma (in less than 1% of cases) is characterized by tumor overproduction of vasoactive intestinal peptide (VIP) that causes profuse watery diarrhea [66].

**Fig. 12:**
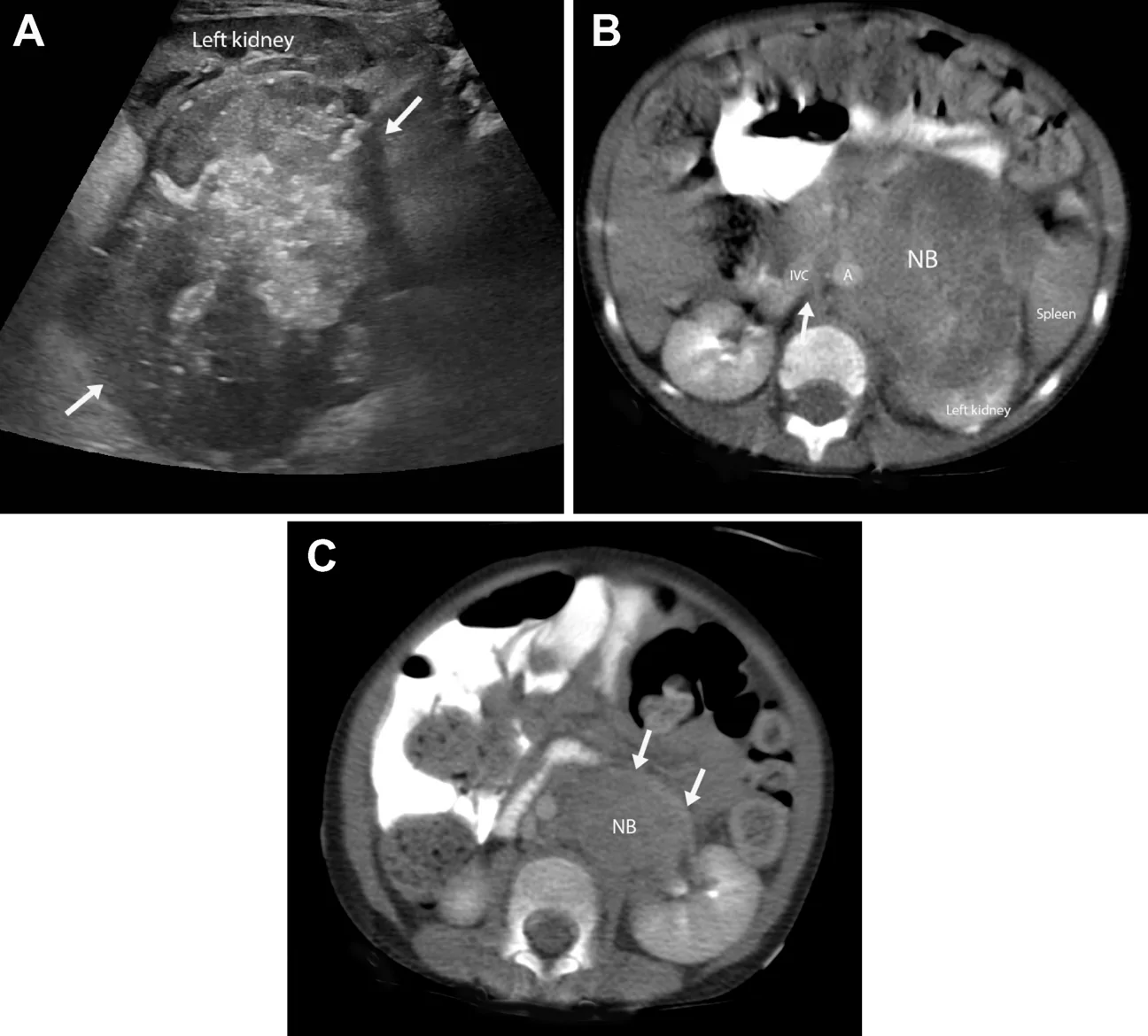
Neuroblastoma diagnosed in a 2-year-old male presenting with a palpable abdominal mass. **a** Abdominal ultrasound in longitudinal plane shows an ill-defined heterogeneous mass (*arrows*) in the left retroperitoneum displacing the left kidney anteriorly. Irregular echogenicities with posterior acoustic shadowing within the mass are consistent with calcifications. **b**, **c** Contrast-enhanced CT of the abdomen in axial plane at the (**b**) mid-level of the mass and (**c**) lower level of the mass shows heterogeneity with non-enhancing, low-attenuating regions consistent with cystic components, possibly necrosis. *NB*, neuroblastoma. Notable image-defined risk factors include neoplasm surrounding the abdominal aorta (*A*) and abutting at least 50% of the surface of the IVC (*arrow*, **b**). The left renal vein is displaced anteriorly and stretched over the ventral aspect of the mass (*arrows*, **c**)

Ultrasound is the initial modality for assessment of suspected intra-abdominal mass in infants and young children [7]. An incidentally discovered, asymptomatic suprarenal neuroblastoma may be small. Most neuroblastomas, however, are large and ill-defined at the time of diagnosis with mass effect upon adjacent structures [58, 67]. If the tumor encroaches upon or invades the renal sinus, differentiating a neuroblastoma from a primary renal tumor can be challenging. Echogenicity is typically heterogeneous. Solid tumor components will be isoechoic to echogenic, and hypoechoic to anechoic necrotic or cystic components may be present as well. Coarse calcifications are present in a high percentage of neuroblastomas, and these create bright echoes with posterior acoustic shadowing (Fig. 12). The site of origin should be attempted, and extension across the midline should be determined [57, 68]. Neuroblastomas tend to displace or encase vessels rather than invade vessels. Grayscale and color Doppler are both useful in evaluating tumor relationship to renal vessels, abdominal aorta, and the IVC. The relationship of tumor to intra-abdominal vessels and other anatomic details is more readily assessed using contrast-enhanced CT and/or MRI (Fig. 12). Both of these cross-sectional modalities are used to determine tumor size, regional extent of disease, and metastatic spread of malignancy [68]. Whereas pulmonary metastases are best assessed on CT imaging, the osseous metastases seen with neuroblastoma are better detected by MRI [57].

Surgical and diagnostic imaging-based staging systems for neuroblastoma have evolved over time. In 2008, the International Neuroblastoma Risk Group Staging System (INRGSS) was developed to create a staging system of disease extent prior to surgical resection [68], using image-defined risk factors (IDRFs) to categorize locoregional (non-metastatic) tumors as L1 (no IDRFs) or L2 (IDRFs present). IDRFs identify imaging surrogates of aggressive tumor growth with respect to local structures and have been validated to predict successful primary tumor resection [59]. Metastatic disease defines INRGSS stage M disease, with a special subcategory for children 18 months or younger with metastases restricted to the skin, liver, and/or limited marrow involvement (stage MS, referring to metastatic, special) [68]. Molecular imaging also contributes to the assessment of neuroblastoma. Iodine-labeled MIBG scintigraphy is used to detect primary tumors and is the test of choice to identify metastatic sites [61, 68–70]. More than 90% of neuroblastomas are MIBG-avid [69]. Non-MIBG-avid disease can instead be evaluated for disseminated disease with ^18^F-FDG PET/CT [57, 71].

Treatment protocols will be driven by the level of risk established [71, 72]. Long-term management of these patients by pediatric endocrinologists is paramount to address sequelae of treatment [73]. Specifically, abdominal radiation therapy and QT alkylating agents increase the risk of gonadal failure, radio-iodine-MIBG therapy increases the risk of thyroid dysfunction and nodules, and surgical resection of an adrenal mass can raise concern for subsequent adrenal insufficiency. These patients are also at increased risk of growth failure and obesity.

### Reproductive systems and endocrinopathies

Abnormalities of the reproductive systems in children commonly require clinical attention by pediatric subspecialists, including urology, gynecology, and endocrinology. Embryologic development of the reproductive systems begins around the sixth week of gestation [74]. Internal genitalia develop in response to the presence or absence of local hormones produced by the ipsilateral gonad, whereas external genitalia develop in response to presence or absence of systemic androgens. Differentiation of the gonads depends on the presence or absence of a Y chromosome [75]. In the presence of the sex-determining region on the Y chromosome, undifferentiated gonads develop into testes. In the absence of a Y chromosome, the gonads differentiate into ovaries. Müllerian-inhibiting factor (MIF), also called anti-Müllerian hormone (AMH), is a glycoprotein secreted by Sertoli cells within the testis that inhibits development of the paramesonephric (Müllerian) ducts. Testosterone produced by Leydig cells in the fetal testes allows the mesonephric (Wolffian) ducts to further develop into the vas deferens, epididymides, and seminal vesicles. In 46,XX fetuses, without circulating androgens and MIF, the Wolffian ducts atrophy while the Müllerian ducts persist and develop into the fallopian tubes, uterus, and the upper vagina. Embryologic developments of the urinary tract and genitalia are inter-related, and therefore renal anomalies such as unilateral renal agenesis are commonly present with genital anomalies [74]. Some of the most commonly seen reproductive system congenital anomalies and acquired abnormalities that involve pediatric radiology and pediatric endocrinology relate to the presentation of primary amenorrhea, which will be reviewed here, with a discussion of appropriate diagnostic imaging and differential considerations.

### Primary amenorrhea

**Fig. 13:**
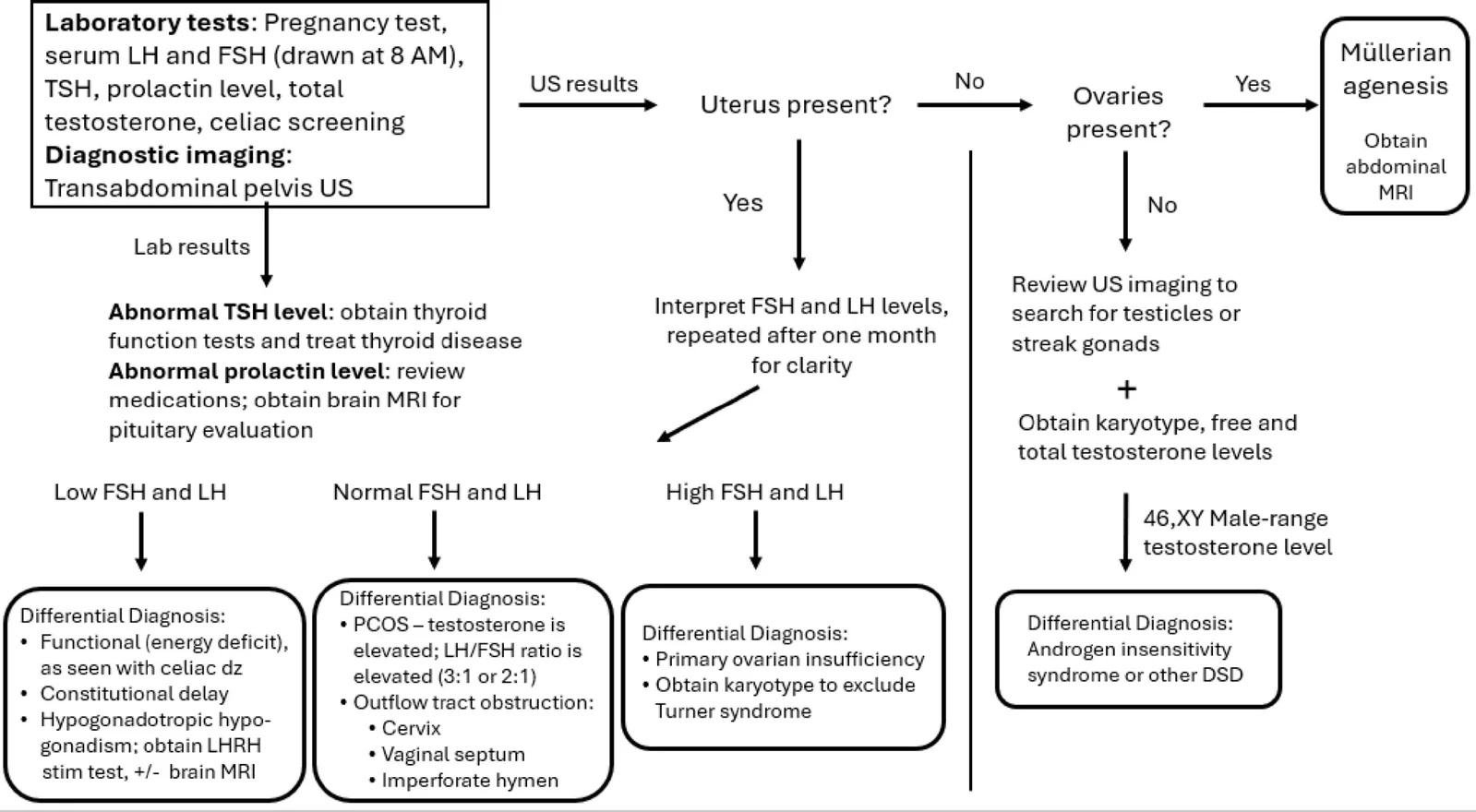
Diagnostic algorithm for primary amenorrhea. Both laboratory data and transabdominal ultrasound guide the diagnosis

The median age of menarche is approximately 12.4 years, and this varies with ethnicity, nutritional status, and weight [76]. Menarche typically begins within 2 years to 3 years of initial breast development (thelarche), which typically occurs around age 10 years. The absence of uterine bleeding in a female of reproductive age is called amenorrhea, which is further classified as primary (no menstruation has ever occurred) or secondary (absence of menstruation for at least 3 months after having had regular menstrual periods previously). Primary amenorrhea is strictly defined as having no menstruation by the age of 15 years or by 3 years after thelarche [76]. This is distinguished from delayed onset of puberty, which applies to females aged 13 years with primary amenorrhea and absence of any secondary sex characteristics, including breast development. The differential diagnosis for primary amenorrhea is broad, but most commonly can be attributed to one of these four etiologies: ovarian insufficiency or dysfunction, Müllerian structural anomalies, hypogonadotropic hypogonadism, or constitutional delay of growth and puberty [77, 78]. Evaluation for primary amenorrhea requires a complete history and physical examination, a urine pregnancy test, serum laboratory tests, and diagnostic imaging of the pelvis beginning with transabdominal ultrasound (see Fig. 13 for a diagnostic algorithm).

**Fig. 14:**
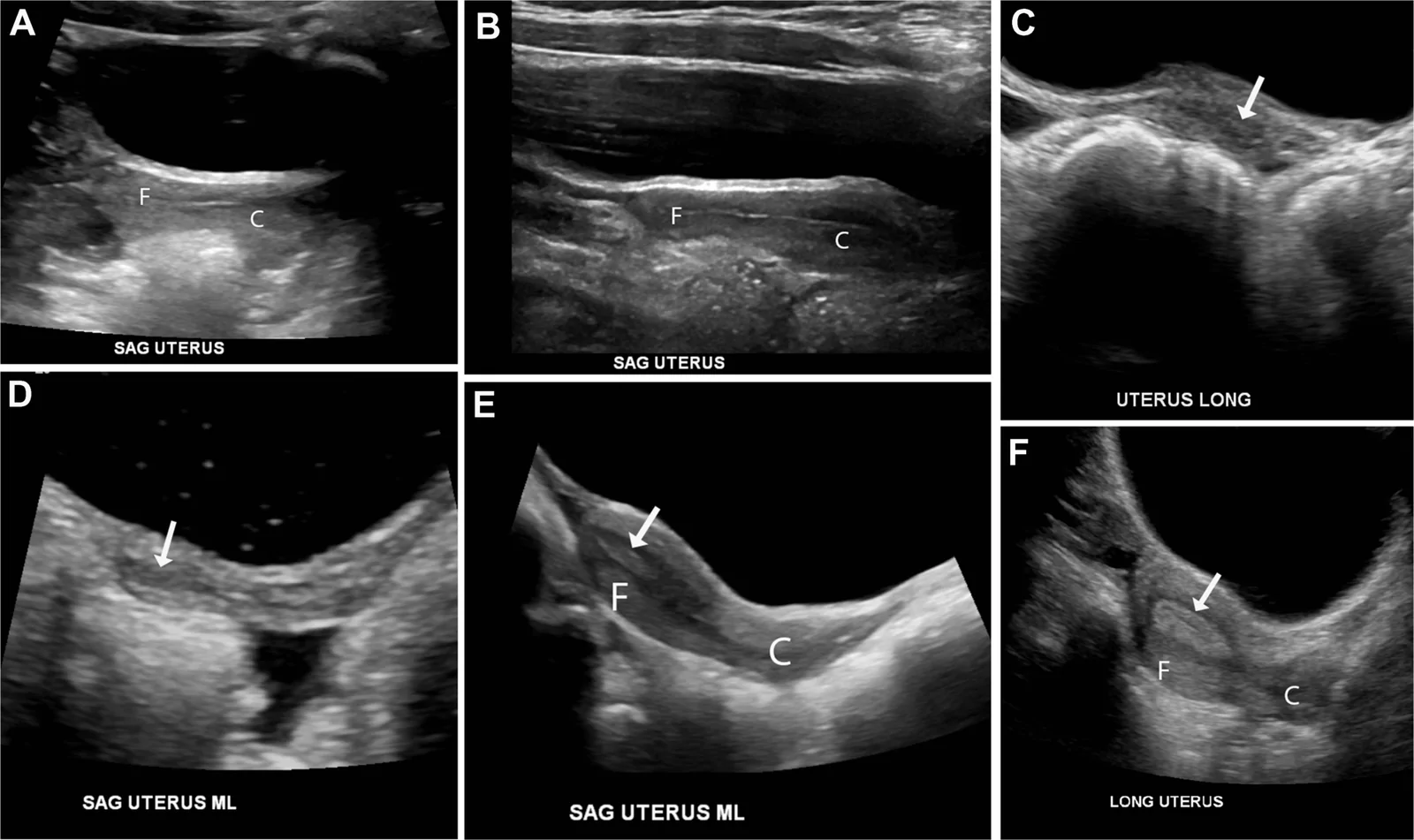
Normal appearances of the uterus over increasing ages. Longitudinal midline transabdominal pelvis ultrasound images show the sagittal appearance the uterus in several different patients at the following ages: (**a**) 6 months (3.2 cm long); (**b**) 9 months (3.2 cm long); (**c**) 4 years (1.8 cm long); (**d**) 9 years (3.2 cm long); (**e**) 12 years (6.8 cm long); and (**f**) 17 years (7.2 cm long). Note that in infancy (**a**, **b**), the uterus has a tubular morphology and the ratio of the fundal (*F*) length to cervical (*C*) length is close to 1:1. In the pre-pubertal school age (**c**, **d**), the uterus is small with only a faintly visible fundal endometrial stripe. At puberty (**e**, **f**), the fundus (*F*) enlarges disproportionate to the cervix (*C*), and the endometrial stripe (*arrow*) thickens

**Fig. 15:**
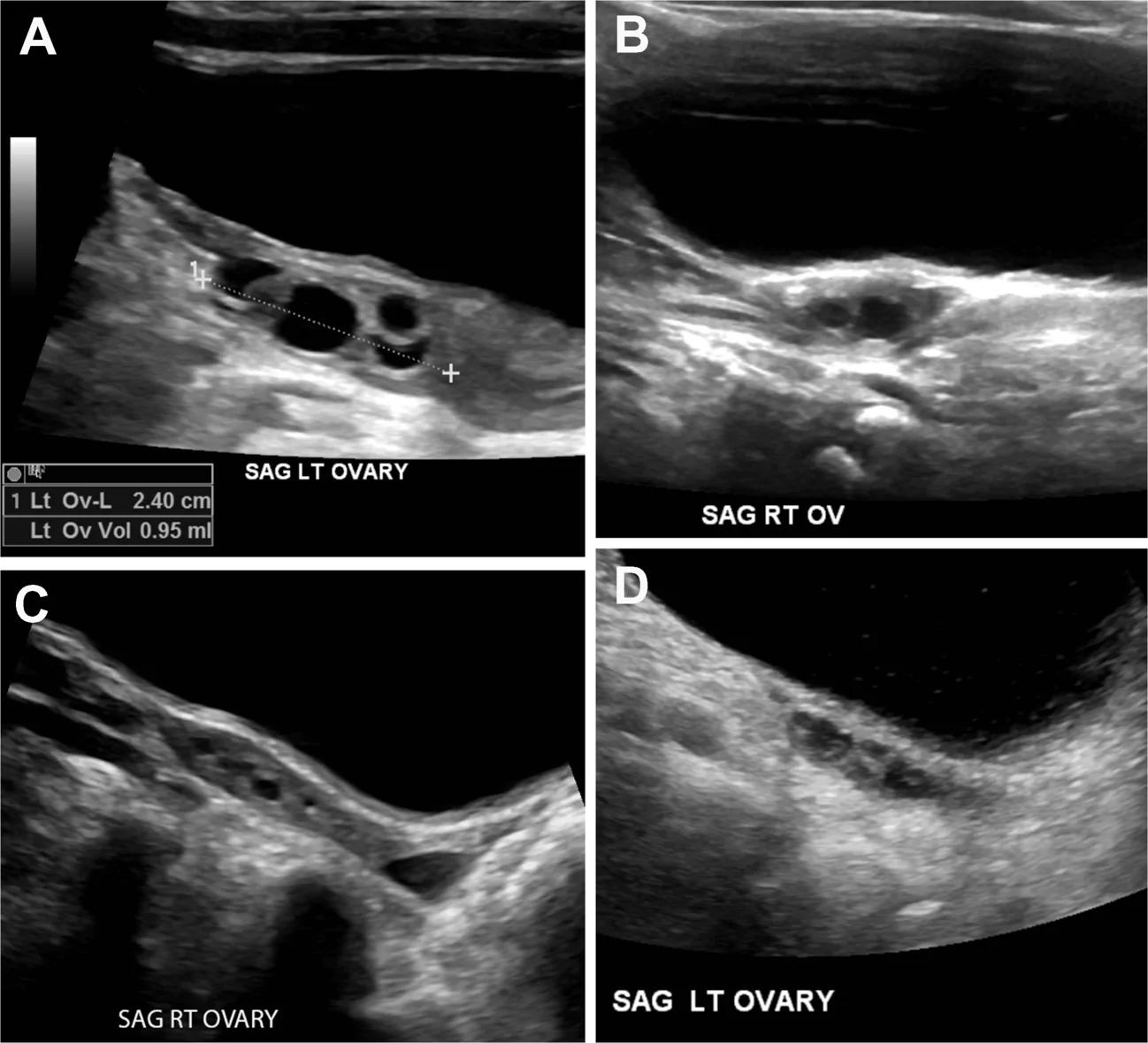
Normal appearance of ovaries in the infant, young child, and postpubertal child. Ultrasound images of normal ovaries show a rounded to oblong appearance of the ovary with several small follicles at the following ages: (**a**) 6 months (0.95 mL); (**b**) 9 months (0.45 mL); (**c**) 4 years (2.7 mL); (**d**) 9 years (4.5 mL)

The purpose of a transabdominal pelvic ultrasound is to identify the presence of a uterus, and its size and morphology, as well as to identify the ovaries and evaluate their appearance. Observed sizes of the uterus and ovaries can be compared with published normal values based on age [79, 80]. Uterine longitudinal length measures approximately 4 cm in the newborn infant and decreases in size between birth and age 4 years before steadily increasing over the premenarchal years [80, 81]. The fundal-cervical length ratio is approximately 1:1 in infancy, and this decreases until age 4 years before gradually increasing to a ratio close to 3:1 by 15 years of age (Fig. 14) [79]. A uterine endometrial echogenic stripe may normally be seen in infants less than 6 months but otherwise may not be appreciable before 12 years of age or until Tanner pubertal stage 2 [79]. Ovaries in the neonate and toddler measure approximately 1 cm^3^, and ovarian volumes increase exponentially between infancy and age 15 years [79, 81, 82]. Ovaries are normally round or oblong in shape, with several follicles measuring up to 7 mm in diameter before age 8 years; after age 8 years, the percentage of females with more than six follicles per ovary increases [82] (Fig. 15). Uterine volume and body length have been shown to correlate best with age and stage of puberty [80].

**Fig. 16:**
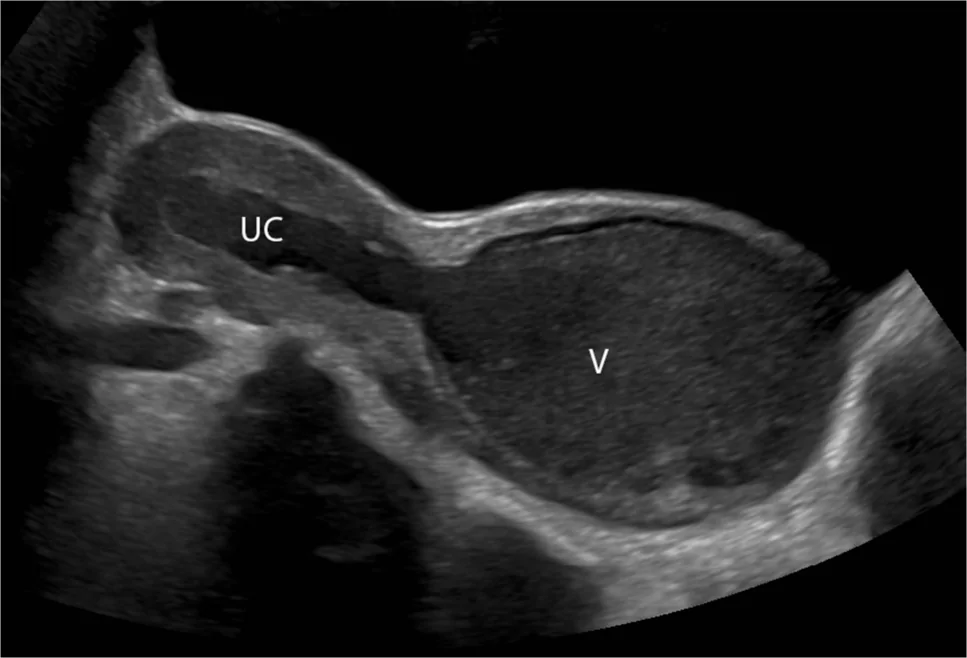
Hematometrocolpos in a 14-year-old with vaginal agenesis. Longitudinal midline ultrasound image through the pelvis shows abnormal distention of the uterine cavity (UC) and vagina (V) by homogenously echogenic material consistent with blood products

If the transabdominal pelvis ultrasound does not provide visualization of a uterus, considerations include Müllerian agenesis and differences of sex development (Fig. 13), discussed below. When a uterus is identified, assessment of the endometrium is important [83]. Distention of the endometrial cavity with heterogeneous material is diagnostic of hematometra, caused by outflow tract obstructions such as imperforate hymen, transverse septum, or vaginal agenesis (Fig. 16). A thin endometrial lining after Tanner stage 2 of puberty is suggestive of a lack of estrogen exposure, whereas a thickened endometrial lining could signify the presence of estrogen but chronic anovulation, assuming there is no uterine or vaginal outflow obstruction. Inability to identify the ovaries on transabdominal ultrasound is concerning for gonadal dysgenesis, most commonly seen in Turner syndrome, which affects one in every 2,500 live female births [84]. If the presence or absence of internal female genitalia cannot be confirmed on ultrasound, a non-contrast pelvis MRI is an appropriate next step (Table 2).

**Table 2:** Pediatric reproductive system disorders that cause endocrine abnormalities and their key imaging features

Uterine and ovarian pathologies			
Müllerian anomalies	• Transabdominal pelvic US is the initial modality to assess internal female reproductive anatomy; transvaginal ultrasound may be used when feasible in adolescents • MRI pelvis without contrast is the gold standard diagnostic technique **MRI pelvis protocol for** **uterine anomalies**: Preparation — when feasible in adolescents, vaginal gel may be introduced before scanning to delineate vaginal canal and outline septa Coronal large FOV T2W SSFSE to include the kidneys Sagittal T2W FSE Coronal oblique T2W 3D FSE angled in-plane with the uterus (long axis) Axial oblique T2W FSE (angled short-axis to the uterus) Axial T2W FSE fat-saturated Axial T1W 2D dual-echo SPGR Axial balanced GRE	• Nine subtypes of uterine anomalies: Müllerian agenesis, cervical agenesis, unicornuate uterus, uterine didelphys, bicornuate uterus, septate uterus, transverse vaginal septum, longitudinal vaginal septum, and complex anomalies • Renal agenesis and renal ectopia are the most common associated congenital anomalies	• Primary amenorrhea • Increased risk of endometriosis, pelvic inflammatory disease, infertility, and birthing complications
Mayer-Rokitansky-Kuster-Hauser (MRKH) syndrome	MRI pelvis without contrast is the gold standard diagnostic technique (same protocol as above for Mullerian anomalies)	• Rudimentary Müllerian structures are more readily assessed on MRI than by US; aplasia of the uterus, cervix, and upper 2/3 of vagina • Associated anomalies are known to occur in some individuals with MRKH syndrome: renal anatomic anomalies, vertebral anomalies, scoliosis and other skeletal anomalies, cardiac anomalies	• Primary amenorrhea
Polycystic ovarian syndrome (PCOS)	• US imaging of ovaries specifically for establishing a diagnosis of PCOS is not recommended until 8 years post-menarche • Transabdominal pelvic US is the initial modality to assess internal female reproductive anatomy; transvaginal ultrasound may be used when feasible in adolescents	At 8 years or more post-menarche, features of PCOS include ovarian volume enlargement and visualization of ≥ 10 mL ovarian volumes or ≥ 10 follicle number per section by transabdominal US	• Ovulatory dysfunction defined as menstrual intervals persistently < 21 or > 45 days after second year post-menarche • Weight gain, insulin resistance, acne, and hirsutism
Disorders of sexual differentiation			
46,XY gonadal dysgenesis	• Transabdominal pelvic ultrasound and abdominopelvic MRI both have limitations in identifying intra-abdominal testes or streak gonads • Surgical laparoscopy facilitates direct inspection of intraperitoneal structures and identification of the gonads	• Absence of the gonads or streak appearance (very small, hypoechoic) of ovotestes on transabdominal US	• Absent to minimal thelarche • Primary amenorrhea
Complete androgen insensitivity syndrome	• Transabdominal pelvic ultrasound is the appropriate initial modality to identify the Müllerian structures and internal gonads • Non-contrast MRI of the pelvis may be necessary to evaluate internal anatomy	• Vaginal pouch with absent Müllerian structures and morphologically normal testes within the inguinal canals or pelvis	• Primary amenorrhea in setting of normal female external genitalia and normal secondary sex characteristics

*FS*, fat-saturation; *FSE*, fast-spin echo; *GRE*, gradient echo; *SPGR*, spoiled dual gradient echo; *SSFSE*, single-shot fast-spin echo; *US*, ultrasound

### Müllerian structures and assessment

Normally by week 6 of embryologic development, the paired Müllerian ducts have formed. Interruption at this early stage results in developmental anomalies including agenesis or hypoplasia of the uterus, fallopian tubes, cervix, upper vagina, and unicornuate uterus [75]. Following normal formation, the paired ducts fuse from their caudal to cranial ends. Interruptions in this process result in fusion anomalies, such as uterine didelphys. The last phase in uterine embryologic development is degeneration of the margin between the paired ducts, termed resorption. Disruption of resorption leads to types of uterovaginal septum [75]. The American Society of Reproductive Medicine classifies nine subtypes of uterine abnormalities (Müllerian agenesis, cervical agenesis, unicornuate uterus, uterine didelphys, bicornuate uterus, septate uterus, transverse vaginal septum, longitudinal vaginal septum, and complex anomalies) [85]. Some Müllerian anomalies (particularly vaginal) may be seen in more than one category. The reader is encouraged to refer to the excellent summary of this classification by Pfiefer et al. which includes an interactive digital tool for diagnosis and treatment recommendations [85].

**Fig. 17:**
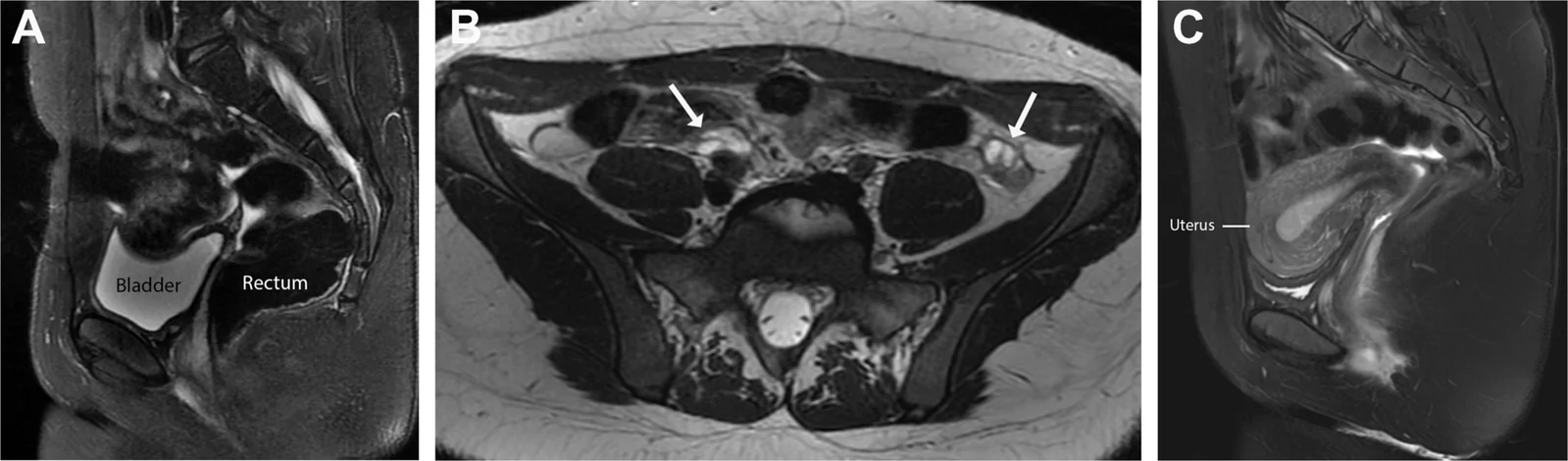
A 14-year-old female with primary amenorrhea and Müllerian agenesis on MRI. **a **Sagittal T2-weighted fat-suppressed image of the midline pelvis shows absence of the uterus posterior to the bladder. **b** Axial T2-weighted image shows presence of bilateral ovaries (*arrows*). **c **In comparison, a sagittal T2-weighted image through the midline pelvis of a normal 17-year-old female shows expected position of an anteverted uterus

In adolescents, Müllerian anomalies can be assessed by two-dimensional and three-dimensional ultrasound and by MRI [74, 86]. If suspicion for Müllerian abnormalities is raised on initial ultrasound imaging, patients should have an MRI including a large field-of-view coronal T2-weighted sequence through the abdomen to visualize the bilateral kidneys (a standard MRI protocol is summarized in Table 2) [87]. Oblique coronal and sagittal T2 sequences are important to distinguish the endometrial and myometrial layers of the uterus (Fig. 17). MRI allows for identification of normal ovaries bilaterally and absence of the uterus and upper two-thirds of the vagina (Fig. 17).

Müllerian agenesis comprises a spectrum of anatomic disorders affecting one in every 4,500-5,000 female live births [88]. The most severe form of Müllerian anomaly is Mayer-Rokitansky-Kuster-Hauser (MRKH) syndrome, characterized by aplasia of the uterus, cervix, and upper two-thirds of the vagina (Fig. 17). Because ovarian function is normal and external genitalia are normal, affected girls usually present during adolescence with primary amenorrhea and normal secondary sex characteristics [75, 88]. Renal agenesis and renal ectopia are the most common associated congenital anomalies [89]. Using MRI, rudimentary Müllerian structures are found in 90% of patients with Müllerian agenesis, whereas by ultrasound, these rudimentary Müllerian structures are difficult to interpret, particularly before puberty [89].

Although MRKH syndrome may manifest as an isolated urovaginal aplasia (MRKH type I), it is important to evaluate for extragenital anomalies (MRKH type II), including renal, skeletal, ear and cardiac anomalies [90]. In one series by Pittock et al. that included 25 patients with MRKH syndrome, the authors observed the following anomalies in their cohort: unilateral renal agenesis (28%), vertebral anomalies (44%), scoliosis (20%), non-vertebral skeletal anomalies (16%), and cardiac anomalies (16%) [89]. In other studies, the frequency of renal anomalies in the setting of MRKH is as high as 40% and may include renal agenesis, ectopia, fusion, dysplasia, malrotation, or duplication [75, 87]. Most cases of MRKH are sporadic; however, familial cases implicating various genetic mutations have been reported [90, 91].

**Fig. 18:**
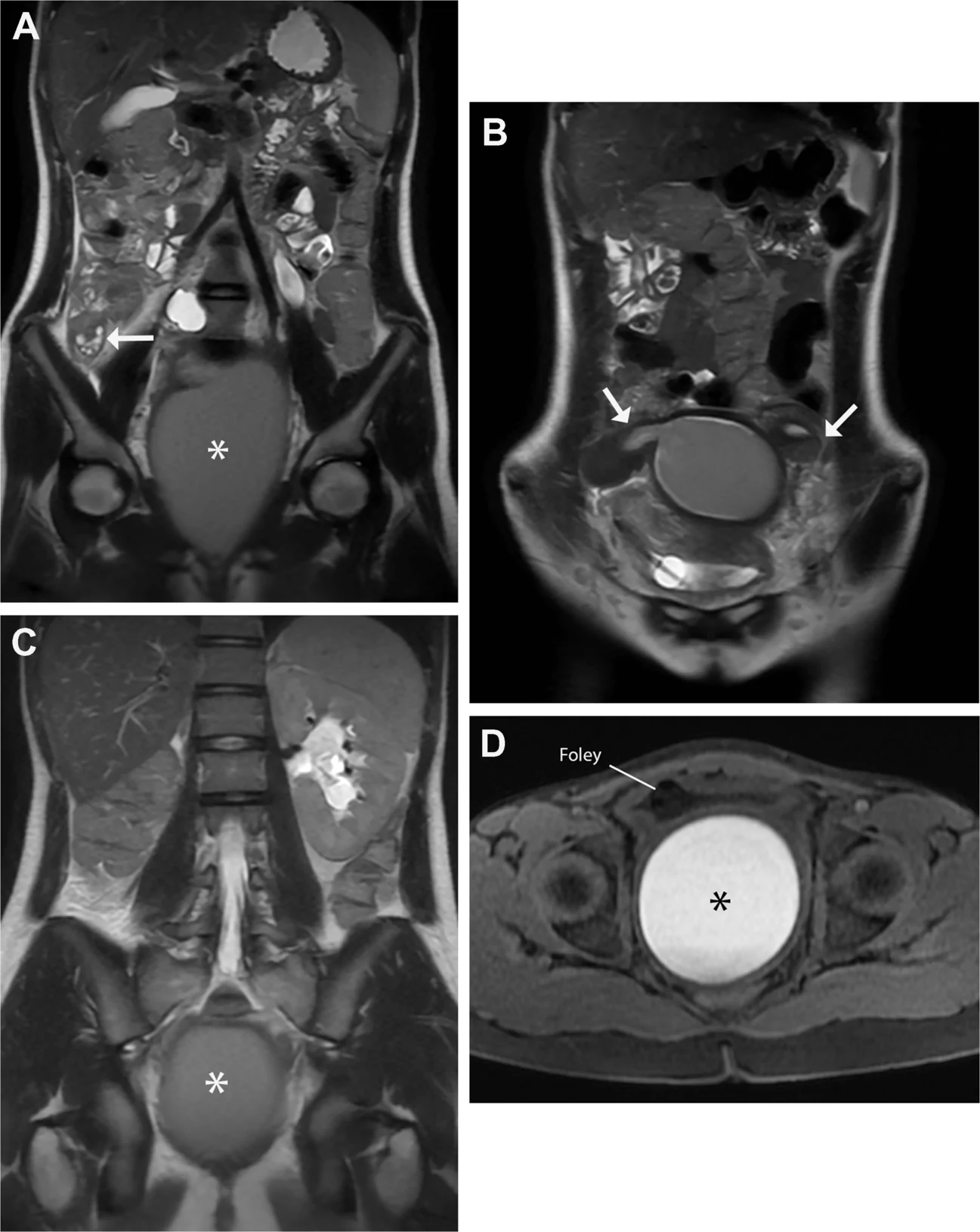
A 14-year-old girl with primary amenorrhea and obstructed hemivagina and ipsilateral renal agenesis (OHVIRA) on MRI. **a**-**c** Coronal T2-weighted images of the abdomen and pelvis and (**d**) axial T1-weighted fat-suppressed image of the lower pelvis show marked distention of the vagina (*asterisk*) by low T2-weighted and high T1-weighted blood products. Uterus is bicornuate, with laterally diverging uterine horns (*arrows*, **b**). Blood products are present in the right uterine horn. Normal ovaries are seen in the far lateral aspects of the pelvis bilaterally (*arrow* points to right ovary in **a**; left not shown). **c** Solitary left kidney with urinary tract dilatation is observed. **d** Distended vagina anteriorly displaces the urinary bladder, decompressed by a Foley catheter

In consideration of other uterine structural anomalies that create outflow obstruction, it is worth mentioning that uterine and vaginal septa may be transverse and obstructing in various configurations of Müllerian anomalies [85]. For instance, in the setting of uterus didelphys and in complex anomalies, transverse septae or noncommunicating hemiuteri will lead to isolation of a uterine cavity with hormonally responsive endometrium. In this scenario, patients may experience cyclical pelvic pain. These patients should be evaluated by MRI for obstructed hemi-vagina and ipsilateral renal agenesis (OHVIRA) syndrome (also known as Herlin–Werner–Wunderlich syndrome) (Fig. 18) [91]. Management is surgical resection of the obstructed hemiuterus or obstructing septum by pediatric gynecology [87, 92].

While primary amenorrhea is the most likely clinical presentation of a uterine anomaly in adolescents, it is worth noting that adolescents with Müllerian anomalies are also at a higher risk of endometriosis and pelvic inflammatory disease. Further gynecological and obstetrical complications of these anomalies, including infertility, miscarriage, elevated risk of ectopic pregnancy, preterm labor, and postpartum hemorrhage, may be additional challenges included in counseling discussions with adolescent patients and families [75, 85, 92].

### XY differences of sex development presenting with amenorrhea

A complete discussion of differences of sex development (DSD) is beyond the scope of this review, but within the differential considerations for primary amenorrhea, 46,XY gonadal dysgenesis and androgen insensitivity syndrome are relevant. When the clinical signs and symptoms point to the possibility of ovarian insufficiency, chromosomal analysis is a key diagnostic test to rule out Turner syndrome and 46,XY gonadal dysgenesis [77]. Individuals with 46,XY gonadal dysgenesis will present with absent or minimal thelarche, normal external female genitalia, and primary amenorrhea. As described earlier, the embryonic development of male genitalia is dependent on testicular hormone production and effective androgen activity. A number of genetic mutations have been identified that disrupt early gonadal development or differentiation, leading to failed testicular development [93]. Instead, Müllerian structures form, and the gonads develop into streak gonads (a term referring to underdeveloped gonads composed of streak-like fibrous tissue) or, very rarely, ovaries or ovotestes. The sonographic appearance of ovotestes has mixed characteristics of both types of gonadal tissue, whereas streak gonads are small and hypoechoic without ovarian stroma or follicles [94]. Ultrasound and MRI are limited in identifying gonads in this setting, although ultrasound has been shown to identify a higher proportion of dysgenetic gonads than MRI [94]. Given the limitations of diagnostic imaging to identify intra-abdominal and dysgenetic gonads, surgical laparoscopy is the most effective method for identifying internal gonads in 46,XY gonadal dysgenesis [95]. Surgical gonadectomy prior to adulthood may be pursued because of the increased risk of malignancy developing within abdominal or inguinal gonads [96]. In some cases, resection may be postponed and surgical relocation of the gonads to the labia/scrotal folds is performed to allow for serial monitoring by clinical exam and ultrasound [94]. Given the typical very small size of streak gonads, visualization of any sizable gonadal tissue in a patient who is expected to have streak gonads should raise suspicion for malignancy [94, 96].

**Fig. 19:**
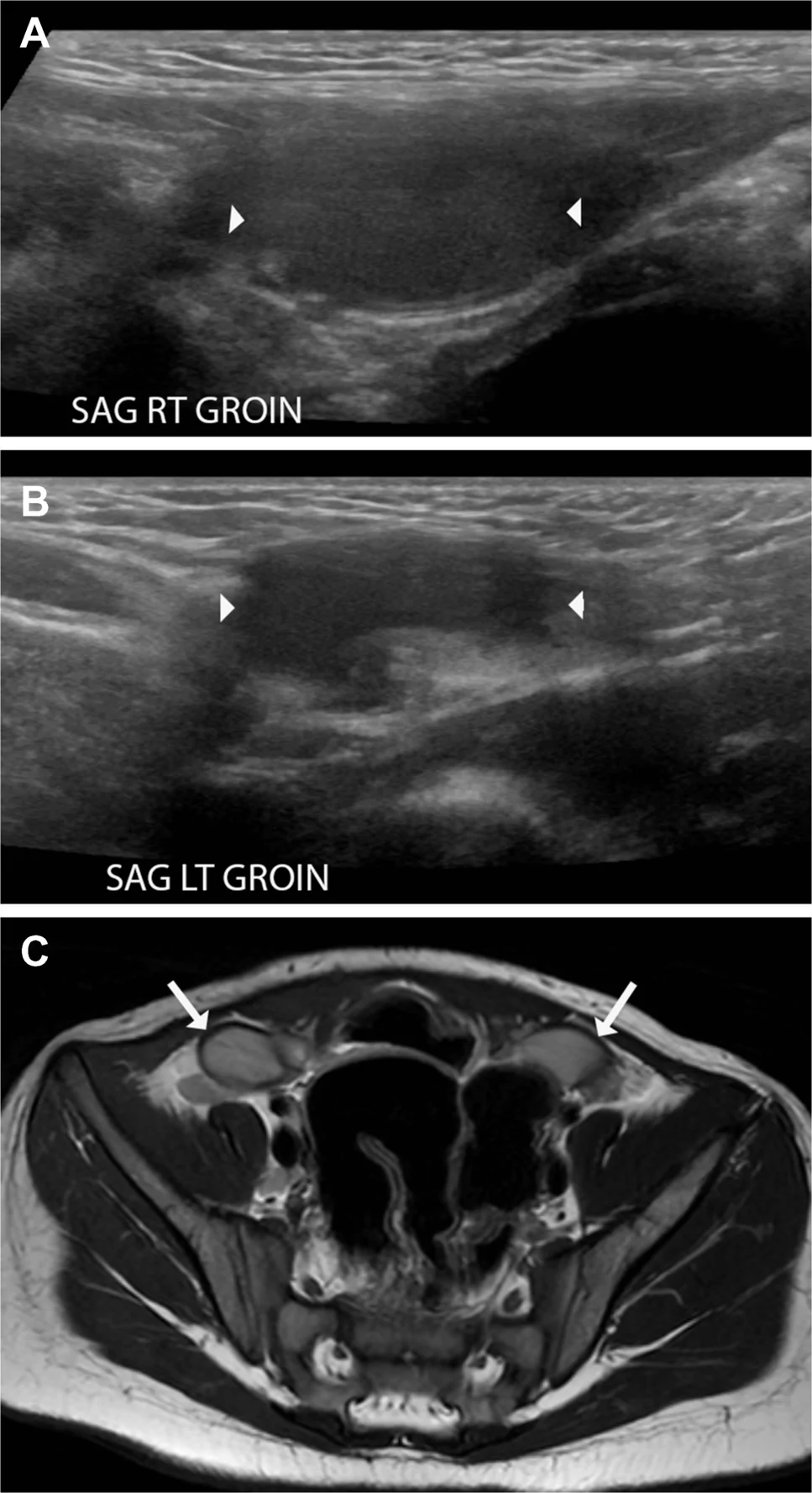
Androgen insensitivity syndrome suspected in a 1-year-old 46,XY infant with normal external female genitalia. Pelvis ultrasound images of the (**a**) right and (**b**) left inguinal regions show oblong homogenous solid tissue within the bilateral inguinal canals most consistent with testicles (*arrowheads*). **c** The gonads resemble testicles in lower pelvis (*arrows*) on subsequent T2-weighted MRI acquired 2 months later. Renal ultrasound shows normal appearances of bilateral adrenal glands and kidneys (*not shown*). No discernible ovaries or uterus was identified by ultrasound or MRI (*not shown*)

In other conditions of 46,XY DSD, the etiology is defective androgen production or defective activity due to gonadotropin resistance, steroidogenic enzyme dysfunction in the adrenal glands, or defects in the androgen receptor. Complete androgen insensitivity syndrome (CAIS), which has a prevalence of one in 25,000 individuals, results in phenotypically normal female external genitalia with abdominal or undescended testes and a lack of Wolffian structures [78, 93]. Because of the intact testicular anti-Müllerian hormone secretion in these individuals, the Müllerian ducts typically (but not invariably or completely) regress leading to absence of the fallopian tubes, uterus, cervix, and upper vagina [97]. The distal vagina, arising from the urogenital sinus, is present as a vaginal pouch [77]. Diagnostic imaging reveals no Müllerian structures, although testes within the inguinal canals may be identified (Fig. 19) [97]. In CAIS, visualized testicles appear similar to those in the normal male, characterized by an oval shape and homogenous, smooth echogenicity. In one small series of androgen insensitivity syndrome cases, a common finding by ultrasound were gonadal nodules characterized by oval, well-demarcated, hypoechoic regions that were proven to be Sertoli hamartomas following biopsy or gonadectomy [98].

### Polycystic ovarian syndrome (PCOS)

**Fig. 20:**
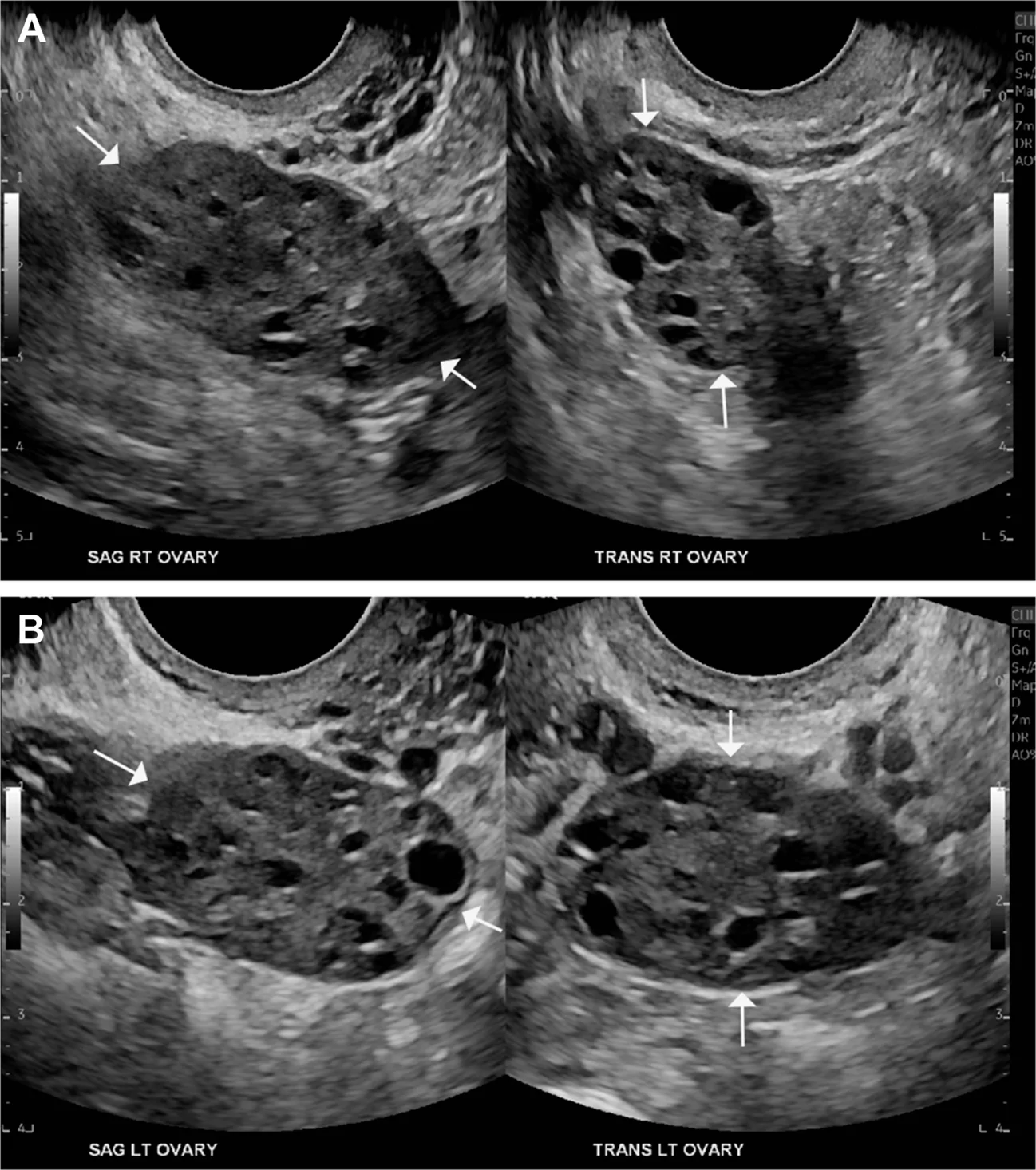
A 20-year-old female with polycystic ovarian syndrome (PCOS), presenting with secondary amenorrhea, obesity, and hirsutism. Transvaginal ultrasound images show abnormally enlarged (**a**) right and (**b**) left ovaries (*arrowheads*) with numerous small follicles typical of PCOS morphology. Right ovarian volume is 18 mL; left ovarian volume is 20 mL

PCOS is a complex multisystem disorder that affects 5–18% of women and classically presents with amenorrhea, weight gain, insulin resistance, acne, and hirsutism [99]. The disorder arises from multiple intersecting sources, including genetic influences, hypothalamic and ovarian dysfunction, excess androgen exposure, and insulin resistance, making the pediatric endocrinologist a central part of management [99, 100]. Diagnostic criteria have been recently refined [100]. Historically, diagnosis has relied on either clinical or laboratory evidence of hyperandrogenism, irregular menstrual cycles, and polycystic ovary morphology based on ultrasound [101]. In adolescents, however, diagnosis is considerably more challenging because of the substantial overlap between normal development and the clinical manifestations of PCOS. For instance, menstrual cycle irregularity, anovulation, and acne as manifestations of hyperandrogenism are all features that typically developing adolescents may manifest, depending on the stage of puberty. Furthermore, a polycystic ovarian morphology (PCOM), characterized by greater than expected ovarian volumes (using a threshold of ≥ 10 mL) or increased follicle number per section (FNPS ≥ 10), has been found in up to one-third of normal patients when assessed at 4 years after the onset of menarche [100, 102] (Fig. 20). For this reason, international guidelines recommend against the use of pelvic ultrasound until 8 years post-menarche [100, 102]. Thus, current diagnostic criteria for PCOS rely on the integration of clinical, laboratory, and imaging clues, including ovulatory dysfunction and clinical or biochemical evidence of hyperandrogenism [100–102]. Transition from adolescent care to adult medicine is important to facilitate, as longitudinal studies suggest that persistent hyperandrogenism during adolescence increases the risk of PCOS in adulthood [100, 103].

## Conclusion

Diagnostic imaging of the abdomen and pelvis with ultrasound, MRI, and sometimes CT for adrenal and reproductive tract abnormalities in children plays a key role in evaluation of challenging cases assessed by the pediatric endocrinologist. Adrenal mass lesions are typically neoplastic, and management relies on a careful assessment for spread of disease with both anatomic and functional diagnostic examinations. The presentation of CAH falls on a spectrum of severity and may present in infancy or later in adolescence, requiring an appreciation of how normal versus hyperplastic adrenal glands appear at different ages. Testicular adrenal rest tumors may occur in males with CAH and can be mistaken for other types of malignancies without an awareness of this association. Reproductive tract abnormalities are most commonly developmental in nature and become apparent during adolescence due to the lack of expected pubertal changes. The clinical scenario of primary amenorrhea in the adolescent female demonstrates the complexity of endocrine function assessment and the importance of pediatric diagnostic imaging in these cases. These diverse scenarios highlight the essential role of pediatric diagnostic imaging in the comprehensive evaluation of complex endocrine and reproductive disorders in children and adolescents.

## Data Availability

No datasets were generated or analysed during the current study.
